# Structure-Based Modeling of SARS-CoV-2 Peptide/HLA-A02 Antigens

**DOI:** 10.3389/fmedt.2020.553478

**Published:** 2020-11-17

**Authors:** Santrupti Nerli, Nikolaos G. Sgourakis

**Affiliations:** ^1^Department of Biomolecular Engineering, University of California, Santa Cruz, Santa Cruz, CA, United States; ^2^Center for Computational and Genomic Medicine, Department of Pathology and Laboratory Medicine, The Children's Hospital of Philadelphia, Philadelphia, PA, United States; ^3^Department of Biochemistry and Biophysics, Perelman School of Medicine, University of Pennsylvania, Philadelphia, PA, United States

**Keywords:** epitope-based vaccine, T cell epitopes, rosetta, SARS-CoV-2, MHC-I, epitope cross-reactivity

## Abstract

SARS-CoV-2-specific CD4 and CD8 T cells have been shown to be present in individuals with acute, mild, and asymptomatic Coronavirus disease (COVID-19). Toward the development of diagnostic and therapeutic tools to fight COVID-19, it is important to predict and characterize T cell epitopes expressed by SARS-CoV-2. Here, we use RosettaMHC, a comparative modeling approach which leverages existing structures of peptide/MHC complexes available in the Protein Data Bank, to derive accurate 3D models for putative SARS-CoV-2 CD8 epitopes. We outline an application of our method to model 8–10 residue epitopic peptides predicted to bind to the common allele HLA-A^*^02:01, and we make our models publicly available through an online database (https://rosettamhc.chemistry.ucsc.edu). We further compare electrostatic surfaces with models of homologous peptide/HLA-A^*^02:01 complexes from human common cold coronavirus strains to identify epitopes which may be recognized by a shared pool of cross-reactive TCRs. As more detailed studies on antigen-specific T cell recognition become available, RosettaMHC models can be used to understand the link between peptide/HLA complex structure and surface chemistry with immunogenicity, in the context of SARS-CoV-2 infection.

## Introduction

An ongoing pandemic caused by the novel SARS coronavirus (SARS-CoV-2) has become the focus of extensive efforts to develop vaccines and antiviral therapies (1). Immune modulatory interferons, which promote a widespread antiviral reaction in infected cells, and inhibition of pro-inflammatory cytokine function through anti-IL-6/IL-6R antibodies, have been proposed as possible COVID-19 therapies (2, 3). However, stimulating a targeted T cell response against specific viral antigens is hampered by a lack of detailed knowledge of the immunodominant epitopes displayed by Human Leukocyte Antigen (HLA) alleles across individuals. The molecules of the class I major histocompatibility complex (MHC-I, or HLA in humans) display on the cell surface a diverse pool of 8–15 amino acid peptides derived from the endogenous processing of proteins expressed inside the cell (4). This MHC-I restriction of peptide antigens provides jawed vertebrates with an essential mechanism for adaptive immunity: surveillance of the displayed peptide/MHC-I (pMHC-I) molecules by CD8 cytotoxic T-lymphocytes allows detection of aberrant protein expression patterns, which signify viral infection and can trigger an adaptive immune response (5). A recent study has shown important changes in T cell compartments during the acute phase of SARS-CoV-2 infection (6), suggesting that a more detailed analysis of antigen-specific T cells would provide new avenues for understanding the expansion and contraction of TCR repertoires in different clinical settings. Given the reduction in breadth and functionality of the naïve T cell repertoire during aging (7), identifying a minimal set of viral antigens that can elicit a protective response will enable the design of diagnostic tools to monitor critical gaps in the T cell repertoire of high-risk cohorts, which can then be addressed using peptide or epitope string DNA vaccines (8).

Human MHC-I molecules are highly polymorphic, with thousands of known alleles in the classical HLA-A, -B, and -C loci. Specific amino acid polymorphisms along the peptide-binding groove (termed A-F pockets) define a repertoire of 10^4^–10^6^ peptide antigens that can be recognized by each HLA allotype (9, 10). Several machine-learning methods have been developed to predict the likelihood that a target peptide will bind to a given allele [reviewed in (11)]. Generally these methods make use of available data sets in the Immune Epitope Database (12) to train artificial neural networks that predict peptide processing, binding and display, and their performance varies depending on peptide length and HLA allele representation in the database. Structure-based approaches have also been proposed to model the bound peptide conformation *de novo* [reviewed in (13)]. These approaches utilize various algorithms to optimize the backbone and side chain degrees of freedom of the peptide/MHC structure according to a scoring function, derived from physical principles (14–16), that can be further enhanced using modified scoring terms (17) or mean field theory (18). While these methods do not rely on large training data sets, their performance is affected by bottlenecks in sampling of different backbone conformations, and any possible structural adaptations of the HLA peptide-binding groove.

Predicting the bound peptide conformation whose N- and C- termini are anchored within a fixed-length groove is a tractable modeling problem that can be addressed using standard comparative modeling approaches (19). For HLA-A^*^02:01, the most common HLA allele (Supplementary Figure 1) among disease-relevant population cohorts (20), there is a large number of high-resolution X-ray structures available in the PDB (21), suggesting that such methods can be applied to produce models of candidate epitopes identified in the proteome of a pathogen of interest. Here, we apply RosettaMHC, a comparative modeling and side chain optimization approach to model all HLA-A^*^02:01 epitopes predicted directly from the ~30 kbp SARS-CoV-2 genome, and make our models publicly available through an online database. Previous studies have shown evidence for T cell cross-reactivity (22, 23) for SARS-CoV-2 viral peptides in healthy individuals (24, 25). Analysis of electrostatic surfaces of our models, relative to models of homologous peptide/HLA-A^*^02:01 complexes derived from four strains of human common cold coronavirus (229E, HKU1, NL63, OC43) allows us to determine epitopes that can elicit SARS-CoV-2 specific and cross-reactive T cell responses. As more data from high-throughput tetramer staining (26–28) and T cell functional screens (29) become available, the models provided here can serve as a toehold for understanding the structural basis of immunogenicity, with actionable value for the development of tetramer-based diagnostics and peptide vaccines to monitor and combat the disease.

## Methods

### Identification of SARS-CoV-2 Peptide Epitopes

The SARS-CoV-2 protein sequences (https://www.ncbi.nlm.nih.gov/nuccore/NC_045512.2) were obtained from NCBI and used to generate all possible peptides of lengths 8, 9, and 10 (9,631 8, 9,621 9, and 9,611 10 mer peptides). We used NetMHCpan-4.0 (30) to derive binding scores to HLA-A^*^02:01, and retained only peptides classified as strong or weak binders [selected using the default percentile (%) rank cut-off values]. The binding classification was performed using eluted ligand likelihood predictions. While in this study we use NetMHCpan-4.0 predictions to select candidate epitopes for structure modeling, our workflow is fully compatible with any alternative epitope prediction method.

### Selection of PDB Templates

To model SARS-CoV-2/HLA-A^*^02:01 antigens, we identified 3D structures from the PDB that can be used as templates for comparative modeling. First, we selected all HLA-A02 X-ray structures that are below 3.5 Å resolution and retained only those that have 100% identity to the HLA-A^*^02:01 heavy chain sequence (residues 1–180). We obtained 236 template structures bound to epitopes of lengths from 8 to 15 residues (of which 1 is an 8 mer, 165 are 9 mers, and 61 are 10 mers). For each SARS-CoV-2 target peptide of (i) length 8, we selected a set of candidate templates of lengths 8–9 by matching the target peptide anchor positions (P1 and P8 in the 8 mer, P2, and P9 in the 9 mer templates), and (ii) lengths 9 and 10, we selected candidate templates of the same peptide length, by matching the target peptide anchor positions (P2 and P9/P10) to each peptide in the template structures. Then, we used the BLOSUM62 (31) substitution matrix to score all remaining positions in the pairwise alignment of the target/template peptide sequences, and the structure with the top score was selected as a template for modeling. For target peptides where we found no templates which matched both peptide anchors, we scored all positions in the pairwise alignment and selected the top scoring template for modeling.

### RosettaMHC Modeling Framework and Database

A detailed description and commands to execute our workflow is available in Supplementary Methods. RosettaMHC (manuscript in preparation) is a comparative modeling protocol developed using PyRosetta (32) to model pMHC-I complexes. The program accepts as input a list of peptide sequences, an HLA allele definition and a template PDB file (selected as described in the previous step). To minimize “noise” in the simulation from regions of the MHC-I structure that do not contribute to peptide binding, only the α_1_ and α_2_ domains are considered in all steps. For each peptide, a full alignment between the target and template peptide/MHC sequences is performed using Clustal Omega (33). The alignment is used as input to Rosetta's threading protocol. From the threaded model, all residues in the MHC-I groove that are within a heavy-atom distance of 3.5 Å from the peptide are subjected to 10 independent all-atom refinement simulations using the FastRelax method (34) and a custom movemap file. Binding energies (dG_separated score terms) are extracted from the refined structures using the interface analyzer protocol. The top three models are selected based on binding energies, and used to compute an average energy for each peptide in the input list. RosettaMHC models of SARS-CoV-2/HLA-A^*^02:01 epitopes are made available through an online database (see Data Availability). The website that hosts our database was constructed using the Django web framework.

### Electrostatic Classification of SARS-CoV-2 Peptide/HLA-A^*^02:01 Complexes

To perform a structure-based classification of SARS-CoV-2 peptide/HLA-A^*^02:01 complexes according to their TCR interaction features and compare their surfaces to homologous peptides from common cold coronavirus strains, we (i) aligned respective protein sequences (specifically, *orf1ab, membrane, spike, envelope*, and *nucleocapsid* proteins) from all strains using Clustal Omega, (ii) extracted 395 (out of 439) epitopes of length 9 from common cold coronavirus strains based on sequence homology with SARS-CoV-2 binders predicted by NetMHCpan-4.0 using default %rank cut-off values (44/439 SARS-CoV-2 epitope sequences are from proteins not considered), (iii) filtered out 141 epitopes containing insertions and deletions in the sequence alignment and those that do not have homologous sequences across all strains of common cold coronaviruses, (iv) modeled structures of the remaining 254 peptide/HLA-A^*^02:01 complexes from each strain using RosettaMHC, and (v) performed a comparison of surface electrostatic potentials between each SARS-CoV-2 pMHC structure and its corresponding common cold coronavirus homologs using multipipsa4.0.2 (35). The multipipsa4.0.2 software applies the Adaptive Poisson-Boltzmann Solver (or APBS) (36) method to first compute electrostatic potentials, and then compares the potentials using the Protein Interaction Property Similarity Analysis (or PIPSA) protocol (37). The side chains of the modeled complexes are protonated using PROPKA (38), followed by assignment of atom charges and radii using the Amber force field (39) at a pH of 7.2. The electrostatic potentials of the structures are calculated by solving a linear Poisson-Boltzmann equation for 129 points on a cubic grid using 150 mM ionic strength at 298.15 Kelvin with protein dielectric of 1.0, and solvent dielectric of 78 using a probe radius of 1.4 Å. Next, the PIPSA protocol compares the electrostatic potentials quantitatively using grid points on the superimposed regions (regions are at a distance of σ from the van der Waals surface and are of thickness δ) around the pMHC complexes. The similarity between any two electrostatic surfaces is captured by the Hodgkin similarity index (HSI, ranges from −1 to 1, where −1, 0, and 1 indicate electrostatic anticorrelation, no correlation, and electrostatic identity respectively) (40), which is converted into a distance measure, D (D = $\sqrt{\left(2-2HSI\right)}$), that assigns values between 0 and 2 (0: identity, 1: no correlation and 2: anticorrelation). For our study, we have used 4 Å thickness (δ) and a distance of 3 Å from the molecular surface (σ) (41).

## Results and Discussion

### Template Identification and Structure Modeling Using RosettaMHC

**Figure 1:**
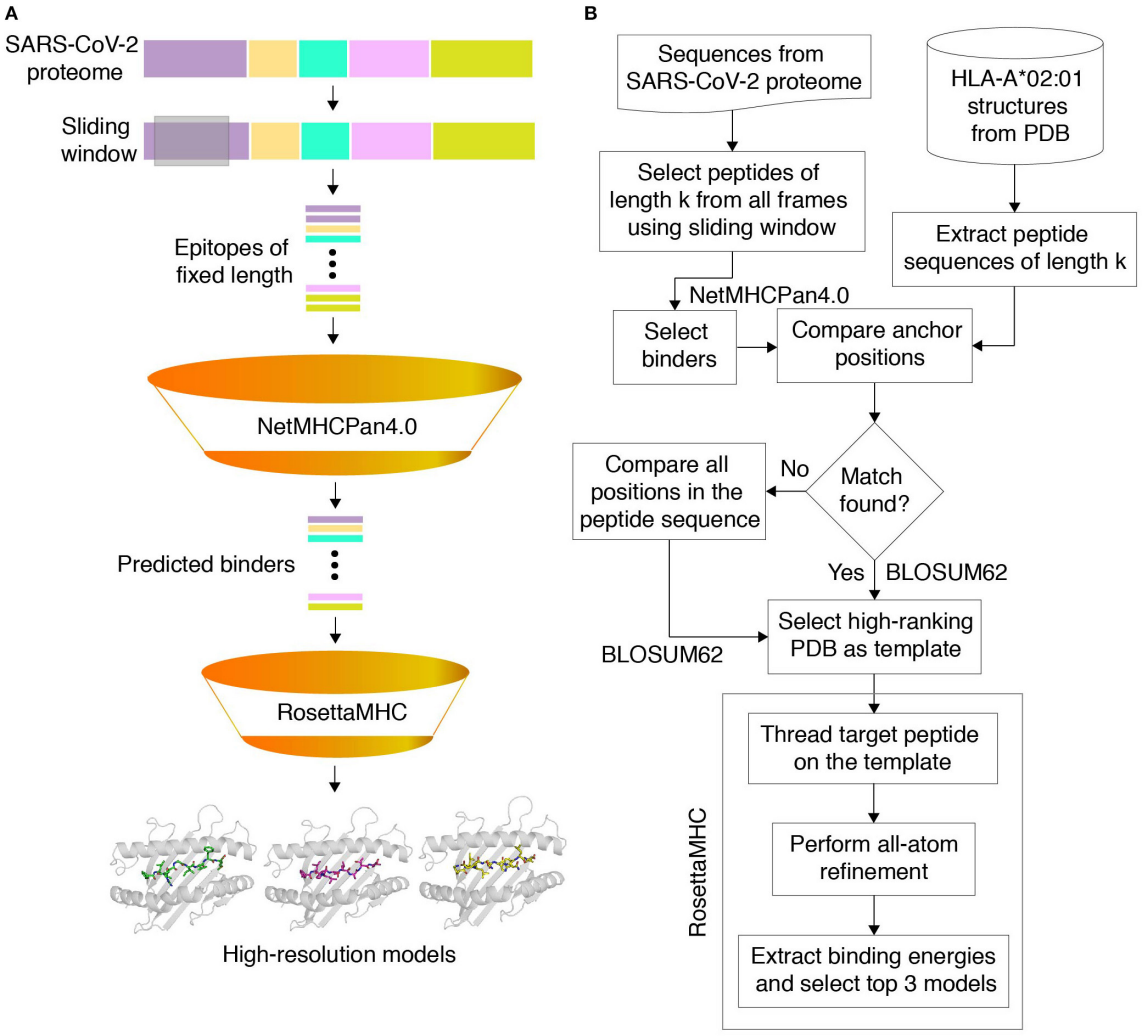
Structure-guided modeling of T cell epitopes in the SARS-CoV-2 proteome. **(A)** General workflow of our pipeline for structure-guided epitope ranking. **(B)** Protein sequences from the annotated SARS-CoV-2 proteome are used to generate peptide epitopes with a sliding window covering all frames of a fixed length (9,631 8, 9,621 9, and 9,611 10 mer possible peptides). Candidate peptides are first filtered by NetMHCpan-4.0 (30) to identify all predicted strong and weak binders (54 8, 439 9, and 256 10 mer epitopes). For rapid template matching and structure modeling, we use a local database of 236 HLA-A*02:01 X-ray structures with resolution below 3.5 Å from the Protein Data Bank (21). Each candidate peptide is scanned against all peptide sequences of the same length in the database, and the top-scoring template is used to guide the RosettaMHC comparative modeling protocol and to compute a binding energy.

Our full workflow for template identification and structure modeling is outlined in Figure 1A, with a flowchart shown in Figure 1B. To predict all possible peptides expressed by SARS-CoV-2 that can bind to HLA-A^*^02:01, we used a recently annotated version of all open reading frames (ORFs) in the viral genome from NCBI (42), made available through the UCSC genome browser (43). We used 8-, 9-, and 10-residue sliding windows to scan all protein sequences, since these are the optimum peptide lengths for binding to the HLA-A^*^02:01 groove (44). The limited availability of templates for peptides of lengths >10 (9 total in the PDB) suggests that such peptides are likely to represent a small fraction of the displayed peptide repertoire, and were not considered here. While spliced peptide epitopes (45) were not considered in the current study, this set can be added to our workflow in future studies. NetMHCpan-4.0 (30) predicted 54 8, 439 9, and 256 10 mer epitopes that can bind to HLA-A^*^02:01 (classified as both weak and strong binders), with the majority of peptides originating from the nsp3 protein encoded by *orf1ab* (NCBI Reference YP_009724389.1) (Supplementary Figure 2). A sequence analysis of all 9 mer peptides predicted by NetMHCpan-4.0 to peptides bound to HLA-A02 structures in PDB showed similar motifs (Supplementary Figure 3). In general, binders predicted by NetMHCpan-4.0 exhibit higher sequence similarity to peptides present in the PDB HLA-A02 structures, relative to non-binders (Supplementary Figure 4).

**Figure 2:**
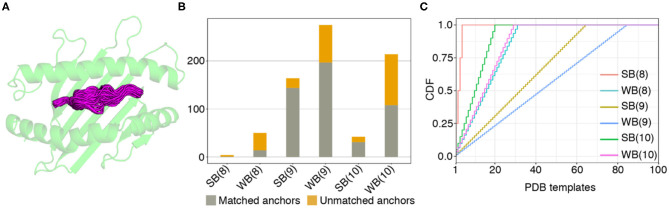
Coverage of predicted HLA-A02 epitopes by structural templates in the PDB. **(A)** Structural overlay of HLA-A02 PDB templates displaying 9-residue peptides, with the different peptide backbone conformations shown in magenta. **(B)** Matching statistics for all predicted SARS-CoV-2 strong (SB) and weak binder (WB) peptides of lengths 8, 9, and 10, against an annotated database of 236 HLA-A02 X-ray structural templates derived from the Protein Data Bank (21). **(C)** Plot showing cumulative distributions (CDF) for strong and weak binder peptides of lengths 8, 9, and 10, as a function of the total number of matching templates used for modeling.

To further validate the NetMHCpan-4.0 predictions and to derive plausible 3D models of the peptide/HLA-A^*^02:01 complexes, we used a structure-guided approach, RosettaMHC, which aims to derive an accurate fitness score for each peptide in the HLA-A^*^02:01 binding groove using an annotated database of high-resolution structures and Rosetta's all-atom energy function (46). RosettaMHC leverages a database of 236 HLA-A^*^02:01 X-ray structures, to find the closest match to each target epitope predicted from the SARS-CoV-2 proteome. Here, the range of available structures in the PDB provides a natural sampling of different possible backbone conformations within the highly restrictive environment of the peptide-binding groove, as shown by a structural alignment of all 9 mer templates (Figure 2A). To identify the best template for modeling of each target peptide, we use sequence matching criteria which first consider the peptide anchors (positions P1/P2/P2 and P8/P9/P10 for 8/9/10 mer epitopes), followed by similarity of the full alignment between the template and target peptide sequences. To demonstrate the accuracy of RosettaMHC, we performed benchmark calculations using a non-redundant set of 90 9-mer peptide/HLA-A02 complex structures. Each epitope was modeled from the closest template with identical anchor residues present in the benchmark set, while homologous peptide sequences were excluded from template selection. From these results, we find that (i) the binding energies of RosettaMHC models fall within the distribution of the native PDB templates (Supplementary Figure 5), and (ii) models generated for 75 and 98% of peptides are within 1.5 and 2 Å backbone heavy-atom RMSD from their native X-ray structures, respectively (Supplementary Figure 5). These results suggest that RosettaMHC can provide accurate models of peptide/HLA-A02 complexes for a range of peptide sequences using a simple threading approach which takes into account the peptide anchor positions as the main criterion for identifying the closest template in the database.

The template assignment statistics for the six different classes of SARS-CoV-2 epitopes in our set are shown in Figure 2B. We find that we can cover the entire set of 749 predicted 8, 9, and 10-residue binders using a subset of 123 HLA-A^*^02:01 templates in our annotated database of PDB-derived structures (Figure 2C). Each target peptide sequence is then threaded onto the backbone of its best identified template, followed by all-atom refinement of the side chain and backbone degrees of freedom using Rosetta's Ref2015 energy function (46), and binding energy calculation.

### RosettaMHC Models Recapitulate Features of High-Resolution X-Ray Structures

**Figure 3:**
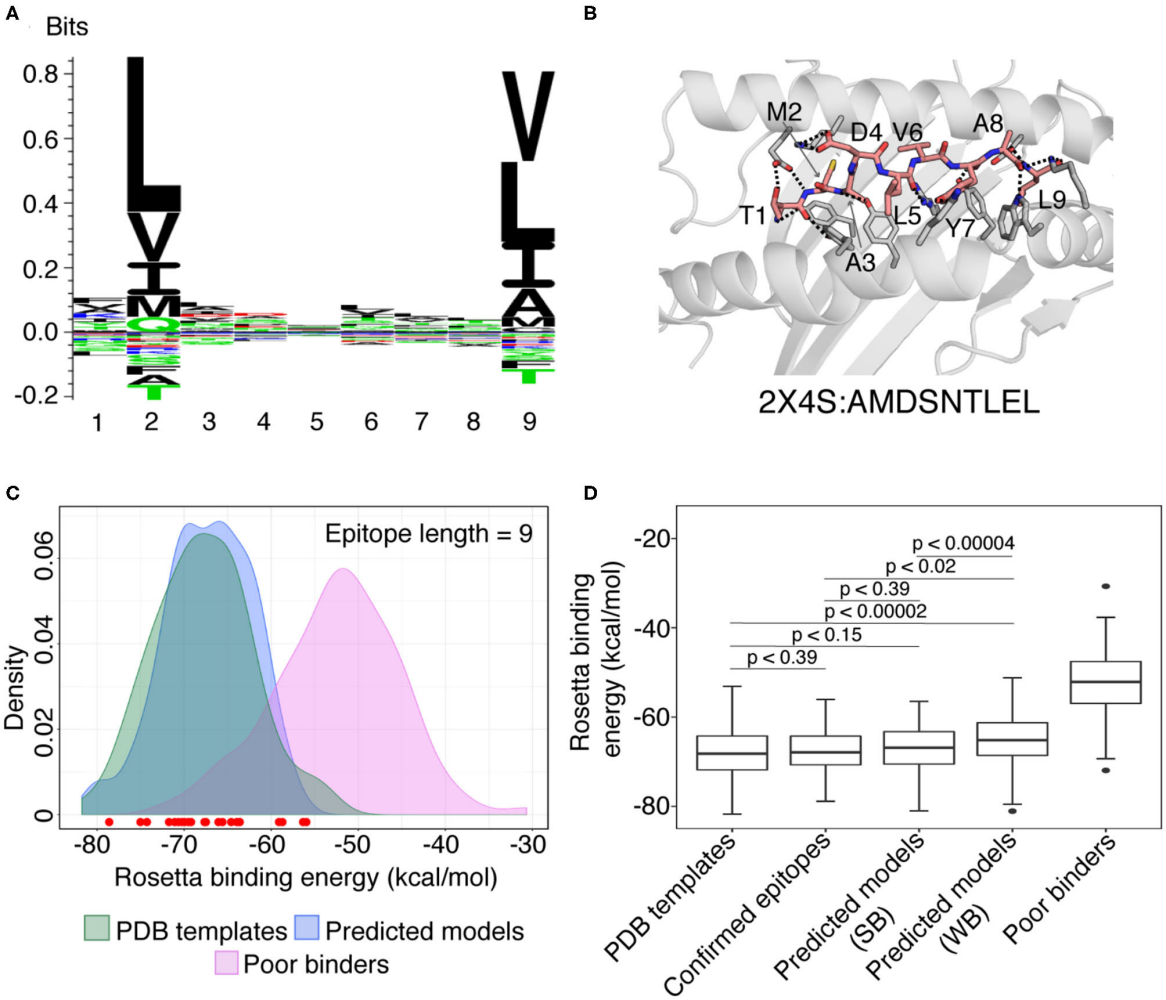
Summary of RosettaMHC modeling results for SARS-CoV-2 peptide epitopes. **(A)** Sequence logo from the 164 top ranking epitopes in the SARS-CoV-2 genome, predicted by NetMHCpan-4.0 (30). **(B)** Model generated for the top 9 mer epitope in our refined set, TMADLVYAL, derived from RNA polymerase. Dotted lines indicate polar contacts between peptide and heavy chain residues, with peptide residues labeled. The template PDB ID and original peptide used for modeling the target peptide is indicated below the model. **(C)** Density plot showing the distribution of average Rosetta binding energies (kcal/mol) for all epitopes of length 9. Distributions reflect 93 PDB templates (green), 164 strong binder epitopes [according to NetMHCpan-4.0 (30)] (blue), and 100 SARS-CoV-2 peptides classified as poor binders by NetMHCpan-4.0 modeled using the PDB templates and used as a reference set for sub-optimal binders (Poor binders; pink). The binding energies of models generated for 28 confirmed SARS T cell epitopes from the IEDB and ViPR (47–49) are indicated by circles at the bottom of the plot. Red circles indicate epitopes that lie within the distribution of refined PDB templates. **(D)** Box plots showing distribution of average binding energies for 93 PDB templates, 100 sub-optimal SARS-CoV-2 peptides, 28 confirmed epitopes (47–49) and RosettaMHC models for 164 strong (SB) and 275 weak (WB) binder 9 mer epitopes predicted from the SARS-CoV-2 proteome using NetMHCpan-4.0 (30). An unpaired Mann-Whitney *U*-test was performed for relevant pairs of distributions and their statistical significance described by the *p*-values (where, *p* < 0.1 is considered statistically significant) are (i) PDB templates and strong binders: *p* < *0.15* (ii) PDB templates and confirmed binders: *p* < *0.39* (iii) PDB templates and weak binders: *p* < *0.00002* (iv) confirmed epitopes and strong binders: *p* < *0.39*, and (v) confirmed epitopes and weak binders: *p* < *0.02*, and (vi) strong and weak binders: *p* < *0.00004*, are shown inside the plot. The sequence logo was generated using Seq2Logo (50).

The sequence logos derived from 9 and 10 mer peptides with good structural complementarity to the HLA-A^*^02:01 groove according to Rosetta's binding energy (see below) adhere to the canonical binding motif, with a preference for hydrophobic, methyl-bearing side chains at the peptide anchor residues P2 and P9/P10 (Figure 3A, Supplementary Figure 6A). In addition, the sequences of high-affinity binders, show preferences for specific amino acids at positions P1, P3, P6/P7, P7/P8 for 9 and 10 mer peptides, respectively (Figure 3A, Supplementary Figure 6A). These preferences are recapitulated in representative 9 and 10 mer models of the two top binders in our set as ranked by Rosetta's energy (Figure 3B, Supplementary Figure 6B), corresponding to epitopes TMADLVYAL and FLFVAAIFYL derived from the RNA polymerase and nsp4 proteins, respectively, both encoded by *orf1ab*. In accordance with features seen in high-resolution structures of HLA-A^*^02:01-restricted epitopes, the peptides adopt an extended, bulged backbone conformation. The free N-terminus of both peptides is stabilized by a network of polar contacts with Tyr 7, Tyr 159, Tyr 171, and Glu 63 in the A- and B- pockets of the HLA-A^*^02:01 groove. The Met (9 mer) or Leu (10 mer) side chain of P2 is buried in a B-pocket hydrophobic cleft formed by Met 45 and Val 67. Equivalently, the C-terminus is coordinated through polar contacts with Asp 77 and Lys 145 from opposite sides of the groove, with the Leu P9/P10 anchor nestled in the F-pocket defined by the side chains of Leu 81, Tyr 116, Tyr 123, and Trp 147. Residues P3-P8 form a series of backbone and side chain contacts with pockets C, D, and E, while most backbone amide and carbonyl groups form hydrogen bonds with the side chains of residues lining the MHC-I groove. These high-resolution structural features are consistent across low-energy models of unrelated target peptides in our input set, suggesting that, when provided with a large set of input templates, a combined threading and side chain optimization protocol can derive accurate models (within 2 Å RMSD), as also shown in our benchmark calculations.

### The Rosetta Energy Function Generally Distinguishes High-Affinity Peptides

To evaluate the accuracy of our models and fitness of each peptide within the HLA-A^*^02:01 binding groove, we computed Rosetta all-atom binding energies across all complexes modeled for different peptide sets. High binding energies can be used as an additional metric to filter low-affinity peptides in the NetMHCpan-4.0 predictions, with the caveat that high energies can be also due to incomplete optimization of the Rosetta energy function as a result of significant deviations between the target and template backbone conformations, not captured by our protocol. We performed 10 independent calculations for each peptide which may allow Rosetta's optimization protocol to sample slight changes in peptide backbone (up to 1 Å from starting structure), and the 3 lower-energy models were selected as the final ensemble and used to compute an average binding energy. The results for all 9 and 10 mer peptides are summarized in Figures 3C,D, Supplementary Figures 6C,D while additional results for 8 mers are provided through our web-interface and outlined in Supplementary Table 1. As a positive binder reference set, we used the binding energies of the idealized and relaxed PDB templates, which are at a local minimum of the Rosetta scoring function. As a reference set for sub-optimal binders, we modeled structures of peptides from SARS-CoV-2 proteome that are classified as poor binders according to NetMHCpan-4.0 (highest %rank values).

We observe a significant, favorable (~−15 kcal/mol) energy gap between the average binding energies computed from the refined PDB templates relative to models obtained for poor binder peptides. The binding energies for all predicted 9 mer and 10 mer binders show a significant overlap with the refined PDB template energies (Figure 3C, Supplementary Figure 6C). Comparison of energy distributions of epitopes that are classified as strong vs. weak binders by NetMHCpan-4.0 shows a moderate bias toward lower binding energies for the strong binders and a larger spread in energies for weak binders, likely due to suboptimal residues at the P2 and P9/P10 anchor positions (Figure 3D, Supplementary Figure 6D, with a significance level, *p* < 0.1 between strong and weak binders for both 9 and 10-mers). As an intendent positive set, we also modeled 28 9 and 5 10 mer peptides that are homologous to peptides in the SARS viral genome and have been previously reported to bind HLA-A^*^02:01 in the IEDB and ViPR (12, 47–49) databases (Supplementary Table 2). Inspection of Rosetta binding energies derived from models in this set shows a similar distribution to the epitopes predicted by NetMHCpan-4.0, with the energies of all the peptides falling well within the distribution of the refined PDB templates (red dots in Figure 3C, Supplementary Figure 6C). Finally, to enrich our set of potential binder peptides, we used a higher NetMHCpan-4.0 cut-off value and modeled structures for 627 additional SARS-CoV-2 epitopes (Supplementary Table 3, Supplementary Figure 7). The analysis of these models is discussed in the Supplementary Results section.

### Comparison of Surface Features of Peptide/HLA-A^*^02:01 Models Relative to Homologous Peptides From Common Cold Coronavirus Strains

Visualization of our models through an interactive online interface provides direct information on SARS-CoV-2 peptide residues that are bulging out of the MHC-I groove, and are therefore accessible to interactions with complementarity-determining regions (CDRs) of T cell receptors (TCRs). Given that αβ TCRs generally employ a diagonal binding mode to engage pMHC-I antigens where the CDR3α and CDR3β TCR loops form direct contacts with key peptide residues (51, 52), knowledge of the surface features for different epitopes allows us to interpret sequence variability between different viral strains. For other important antigens with known structures in the PDB, such features can be derived from an annotated database connecting pMHC-I/TCR co-crystal structures with biophysical binding data (53), and were recently employed in an artificial neural network approach to predict the immunogenicity of different HLA-A^*^02:01 bound peptides in the context of tumor neoantigen display (54). The electrostatic compatibility between self vs. foreign HLA surfaces has been shown to define antibody alloimmune responses (41). Given that antibodies and TCRs use a common fold and similar binding mode to engage pMHC-I molecules (51), surface electrostatic features also play an important role in recognition of peptide/HLA surfaces by their cognate TCRs in the context of SARS-CoV-2 infection.

**Figure 4:**
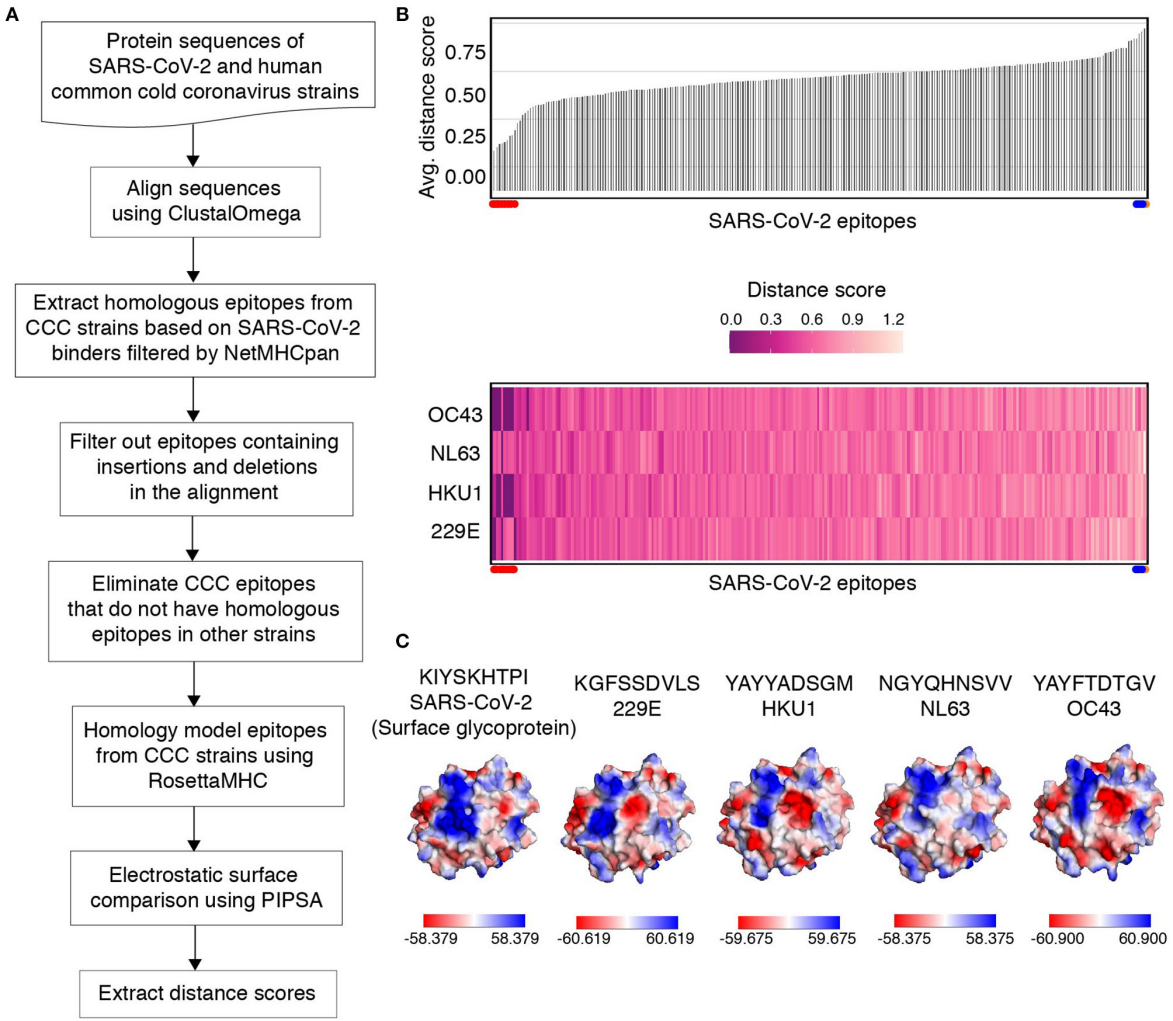
Electrostatic surface similarity scores of SARS-CoV-2 epitopes to peptides derived from four common cold coronavirus strains. **(A)** Flow diagram outlining the comparison of pMHC models for SARS-CoV-2 epitopes, relative to their homologous epitopes in four human Common Cold Coronavirus (CCC) strains based on model electrostatic surface potentials. **(B)** A bar plot (top) showing the average electrostatic similarity score obtained using PIPSA (37) (Y-axis) measured between SARS-CoV-2 epitopes and their homologous common cold coronavirus peptides. Distance scores range between 0 and 2, where 0 indicates electrostatic identity, 1 indicates no correlation and 2 indicates electrostatic anticorrelation. A heat map (bottom) showing individual distance scores between SARS-CoV-2 epitopes (X-axis) and homologous peptides from each strain of common cold coronavirus (Y-axis). The distance scores are indicated by the colored scale (from 0 to 1.2). The SARS-CoV-2 epitopes SLAIDAYPL, AIMTRCLAV, YLGGMSYYC, FVDGVPFVV, RIIPARARV, RILGAGCFV, RLANECAQV, SVFNICQAV, IFVDGVPFV, GVAPGTAVL that share similar electrostatic surfaces with homologous peptides from common cold coronaviruses are highlighted using red dots on the left in top and bottom plots. Similarly, epitopes ALLSDLQDL, QLNRALTGI, MLAKALRKV (blue dots) and KIYSKHTPI (orange dot) that exhibit no apparent correlation or are electrostatically dissimilar are shown on the right in top and bottom plots. **(C)** Structure diagram of SARS-CoV-2 epitope, KIYSKHTPI from surface/spike glycoprotein, showing the maximum electrostatic surface distance to homologous coronavirus epitopes (orange dot in **B**). Homologous peptide sequences in each strain are indicated in each plot. Solvent-accessible surfaces with electrostatic potential representations in the indicated ranges [down to −61 kcal/(mol·*e*) in red and up to +61 kcal/(mol·*e*) in blue] were calculated using the APBS solver (57) in multipipsa4.0.2 (35). All calculations were performed at 150 mM ionic strength, 298.15 Kelvin, pH 7.2, protein dielectric 1.0, and solvent dielectric 78 with a probe radius of 1.4 Å. Graphics were generated using Pymol (58).

T-cell responses to megapools of viral peptides have been observed in individuals not exposed to SARS-CoV-2, thus providing evidence for cross-reactivity of T cells with similar epitopes expressed by homologous coronavirus strains (55, 56). To characterize SARS-CoV-2 specific and cross-reactive epitopes (according to this definition), we obtained homologous peptide sequences from four human common cold coronavirus strains with annotated genomes (229E, HKU1, NL63, and OC43), for all 395 predicted SARS-CoV-2 strong binders of length 9. From this set, 141 peptides are exclusive to SARS-CoV-2, since there are no homologous sequences present in the four common coronavirus strains considered here, or the corresponding sequences in the other four genomes have insertions or deletions (Supplementary Table 5). To identify cross-reactivity according to molecular surface features, we first used RosettaMHC to model peptide/HLA-A^*^02:01 complex structures for all homologous peptides, in addition to our previously described models for SARS-CoV-2 (Figure 4A). We then computed surface electrostatic potentials for each model using APBS (36), followed by a pairwise comparison of potentials computed for the four homologous structures relative to each SARS-CoV-2 peptide using PIPSA (37), which provides four distance scores for each peptide (Figure 4B). From the examination of similarity scores of models, we found that (i) peptide SLAIDAYPL from *orf1ab* has conserved sequence and surface features across all coronavirus strains (distance score = 0), and therefore T cells specific for this epitope should be highly cross-reactive across different strains (ii) epitopes AIMTRCLAV, YLGGMSYYC, FVDGVPFVV, RIIPARARV, RILGAGCFV, RLANECAQV, SVFNICQAV, IFVDGVPFV, and GVAPGTAVL from *orf1ab* are conserved with one or more common strains, and are putatively cross-reactive (distance score ≤ 0.3) (Figure 4B, Supplementary Table 4), and (iii) there is no apparent correlation between SARS-CoV-2 and common cold coronavirus pMHC surface features for ALLSDLQDL (*orf1ab*), QLNRALTGI (*spike*), MLAKALRKV (*orf1ab*), and KIYSKHTPI (*spike*) epitopes (distance score > 0.8) (Supplementary Table 4). In particular, peptide KIYSKHTPI shows the most dissimilar electrostatic surface to the homologous strains among all high-affinity binders, suggesting that this epitope can be used to detect exclusive TCRs to SARS-CoV-2 (Figure 4C). The six epitopes in our set that are known to induce CD8 T cell responses in COVID-19 patients and healthy donors have distance scores ranging from 0.5 to 0.9 (Supplementary Table 6) (24, 25), suggesting that their TCRs can cross-react with homologous epitopes from common cold coronaviruses.

Electrostatic potentials calculated from our models further allow us to compare distinct surfaces for TCR recognition between different high-affinity epitopes, as demonstrated for the four top-scoring models by Rosetta binding energy (Supplementary Figure 8A). Here, PIPSA analyses of electrostatic potentials of these models allowed us to cluster them into two groups, (i) TMADLVYAL and NLIDSYFVV, and (ii) KLWAQVCQL and FLAFVVFLL, where the surface exposed residues at P2-P8 positions of the (i) and (ii) groups exhibit moderately negative and positive charges, respectively (Supplementary Figure 8). Full classification and ranking of all binders in our set on the basis of their molecular surface features would further enable the selection of a diverse panel of peptides for high-throughput pMHC tetramer library generation which can be used to identify immunodominant epitopes (28). Tetramer analysis of T cells from COVID-19 patients, recovered individuals, and healthy donors can help identify critical gaps in the T cell repertoire of high-risk groups, toward the design of epitope DNA strings for vaccine development.

## Data Availability Statement

An online web-interface for visualization and download of all the models is available at: https://rosettamhc.chemistry.ucsc.edu. The RosettaMHC source code is available at https://github.com/snerligit/mhc-pep-threader. Rosetta binding energies for all 749 HLA A^*^02:01-restricted peptides in our set are provided in Supplementary Table 1.

## Author Contributions

SN and NS conceptualized and designed the research. SN performed Rosetta comparative modeling simulations and binding energy calculations, analyzed, and interpreted data. All authors contributed to the article and approved the submitted version.

## Conflict of Interest

The authors declare that the research was conducted in the absence of any commercial or financial relationships that could be construed as a potential conflict of interest.

## References

[1] Liu C Zhou Q Li Y Garner LV Watkins SP Carter LJ. Research and development on therapeutic agents and vaccines for COVID-19 and related human coronavirus diseases. ACS Cent Sci. (2020) 6:315–31. 10.1021/acscentsci.0c00272 32226821 PMC10467574

[2] Kishimoto T. Interleukin-6: discovery of a pleiotropic cytokine. Arthritis Res Ther. (2006) 8:S2. 10.1186/ar1916 16899106 PMC3226075

[3] Kumaki Y Ennis J Rahbar R Turner JD Wandersee MK Smith AJ. Single-dose intranasal administration with mDEF201 (adenovirus vectored mouse interferon-alpha) confers protection from mortality in a lethal SARS-CoV BALB/c mouse model. Antiviral Res. (2011) 89:75–82. 10.1016/j.antiviral.2010.11.007 21093489 PMC3018546

[4] Rock KL Reits E Neefjes J. Present yourself! by MHC Class I and MHC Class II molecules. Trends Immunol. (2016) 37:724–37. 10.1016/j.it.2016.08.010 27614798 PMC5159193

[5] Kaufman J. Unfinished business: evolution of the mhc and the adaptive immune system of jawed vertebrates. Annu Rev Immunol. (2018) 36:383–409. 10.1146/annurev-immunol-051116-052450 29677478

[6] Thevarajan I Nguyen THO Koutsakos M Druce J Caly L van de Sandt CE. Breadth of concomitant immune responses prior to patient recovery: a case report of non-severe COVID-19. Nat Med. (2020) 26:453–5. 10.1038/s41591-020-0819-2 32284614 PMC7095036

[7] Goronzy JJ Fang F Cavanagh MM Qi Q Weyand CM. Naïve T cell maintenance and function in human aging. J Immunol Baltim Md. (2015) 194:4073–80. 10.4049/jimmunol.1500046 25888703 PMC4452284

[8] Oyarzun P Kobe B. Computer-aided design of T-cell epitope-based vaccines: addressing population coverage. Int J Immunogenet. (2015) 42:313–21. 10.1111/iji.12214 26211755

[9] Wooldridge L Ekeruche-Makinde J van den Berg HA Skowera A Miles JJ Tan MP. A single autoimmune T cell receptor recognizes more than a million different peptides. J Biol Chem. (2012) 287:1168–77. 10.1074/jbc.M111.289488 22102287 PMC3256900

[10] Birnbaum ME Mendoza JL Sethi DK Dong S Glanville J Dobbins J. Deconstructing the peptide-MHC specificity of T cell recognition. Cell. (2014) 157:1073–87. 10.1016/j.cell.2014.03.047 24855945 PMC4071348

[11] Peters B Nielsen M Sette A. T cell epitope predictions. Annu Rev Immunol. (2020) 38:123–45. 10.1146/annurev-immunol-082119-124838 32045313 PMC10878398

[12] Vita R Mahajan S Overton JA Dhanda SK Martini S Cantrell JR. The Immune Epitope Database (IEDB): 2018 update. Nucleic Acids Res. (2019) 47:D339–43. 10.1093/nar/gky1006 30357391 PMC6324067

[13] Antunes DA Abella JR Devaurs D Rigo MM Kavraki LE. Structure-based methods for binding mode and binding affinity prediction for peptide-MHC complexes. Curr Top Med Chem. (2018) 18:2239–55. 10.2174/1568026619666181224101744 30582480 PMC6361695

[14] Yanover C Bradley P. Large-scale characterization of peptide-MHC binding landscapes with structural simulations. Proc Natl Acad Sci USA. (2011) 108:6981–6. 10.1073/pnas.1018165108 21478437 PMC3084072

[15] King C Garza EN Mazor R Linehan JL Pastan I Pepper M. Removing T-cell epitopes with computational protein design. Proc Natl Acad Sci USA. (2014) 111:8577–82. 10.1073/pnas.1321126111 24843166 PMC4060723

[16] Liu T Pan X Chao L Tan W Qu S Yang L. Subangstrom accuracy in pHLA-I modeling by Rosetta FlexPepDock refinement protocol. J Chem Inf Model. (2014) 54:2233–42. 10.1021/ci500393h 25050981

[17] Kyeong H-H Choi Y Kim H-S. GradDock: rapid simulation and tailored ranking functions for peptide-MHC Class I docking. Bioinformatics. (2018) 34:469–76. 10.1093/bioinformatics/btx589 28968726

[18] Rubenstein AB Pethe MA Khare SD. MFPred: rapid and accurate prediction of protein-peptide recognition multispecificity using self-consistent mean field theory. PLoS Comput Biol. (2017) 13:e1005614. 10.1371/journal.pcbi.1005614 28650961 PMC5507473

[19] Song Y DiMaio F Wang RY-R Kim D Miles C Brunette T. High-resolution comparative modeling with RosettaCM. Structure. (2013) 21:1735–42. 10.1016/j.str.2013.08.005 24035711 PMC3811137

[20] Robinson J Guethlein LA Cereb N Yang SY Norman PJ Marsh SGE. Distinguishing functional polymorphism from random variation in the sequences of >10,000 HLA-A, -B and -C alleles. PLoS Genet. (2017) 13:e1006862. 10.1371/journal.pgen.1006862 28650991 PMC5507469

[21] Berman HM Westbrook J Feng Z Gilliland G Bhat TN Weissig H. The protein data bank. Nucleic Acids Res. (2000) 28:235–42. 10.1093/nar/28.1.235 10592235 PMC102472

[22] Borbulevych OY Piepenbrink KH Gloor BE Scott DR Sommese RF Cole DK. T cell receptor cross-reactivity directed by antigen-dependent tuning of peptide-MHC molecular flexibility. Immunity. (2009) 31:885–96. 10.1016/j.immuni.2009.11.003 20064447 PMC3248800

[23] Yin Y Mariuzza RA. The multiple mechanisms of T cell receptor cross-reactivity. Immunity. (2009) 31:849–51. 10.1016/j.immuni.2009.12.002 20064442

[24] Chour W Xu AM Ng AHC Choi J Xie J Yuan D. Shared antigen-specific CD8+ T cell responses against the SARS-COV-2 spike protein in HLA A^*^02:01 COVID-19 participants. medRxiv. (2020) 2020.05.04.20085779. 10.1101/2020.05.04.20085779

[25] Nelde A Bilich T Heitmann JS Maringer Y Salih HR Roerden M. SARS-CoV-2 T-cell epitopes define heterologous and COVID-19-induced T-cell recognition. Res Sq Prepr. (2020) 10.21203/rs.3.rs-35331/v132999467

[26] Bentzen AK Marquard AM Lyngaa R Saini SK Ramskov S Donia M. Large-scale detection of antigen-specific T cells using peptide-MHC-I multimers labeled with DNA barcodes. Nat Biotechnol. (2016) 34:1037–45. 10.1038/nbt.3662 27571370

[27] Saini SK Tamhane T Anjanappa R Saikia A Ramskov S Donia M. Empty peptide-receptive MHC class I molecules for efficient detection of antigen-specific T cells. Sci Immunol. (2019) 4:eaau9039. 10.1126/sciimmunol.aau9039 31324690

[28] Overall SA Toor JS Hao S Yarmarkovich M Sara M O'Rourke Morozov GI. High throughput pMHC-I tetramer library production using chaperone-mediated peptide exchange. Nat Commun. (2020) 11:1909. 10.1038/s41467-020-15710-1 32312993 PMC7170893

[29] Ishizuka J Grebe K Shenderov E Peters B Chen Q Peng Y. Quantitating T Cell cross-reactivity for unrelated peptide antigens. J Immunol. (2009) 183:4337–45. 10.4049/jimmunol.0901607 19734234 PMC2762195

[30] Jurtz V Paul S Andreatta M Marcatili P Peters B Nielsen M. NetMHCpan-4.0: improved peptide-MHC Class I interaction predictions integrating eluted ligand and peptide binding affinity data. J Immunol Baltim Md. (2017) 199:3360–8. 10.4049/jimmunol.1700893 28978689 PMC5679736

[31] Henikoff S Henikoff JG. Amino acid substitution matrices from protein blocks. Proc Natl Acad Sci USA. (1992) 89:10915–9. 10.1073/pnas.89.22.10915 1438297 PMC50453

[32] Chaudhury S Lyskov S Gray JJ. PyRosetta: a script-based interface for implementing molecular modeling algorithms using Rosetta. Bioinformatics. (2010) 26:689–91. 10.1093/bioinformatics/btq007 20061306 PMC2828115

[33] Sievers F Wilm A Dineen D Gibson TJ Karplus K Li W. Fast, scalable generation of high-quality protein multiple sequence alignments using Clustal Omega. Mol Syst Biol. (2011) 7:539. 10.1038/msb.2011.75 21988835 PMC3261699

[34] Tyka MD Keedy DA André I Dimaio F Song Y Richardson DC. Alternate states of proteins revealed by detailed energy landscape mapping. J Mol Biol. (2011) 405:607–18. 10.1016/j.jmb.2010.11.008 21073878 PMC3046547

[35] Tong R Wade RC Bruce NJ. Comparative electrostatic analysis of adenylyl cyclase for isoform dependent regulation properties. Proteins Struct Funct Bioinforma. (2016) 84:1844–58. 10.1002/prot.25167 27667304

[36] Baker NA Sept D Joseph S Holst MJ McCammon JA. Electrostatics of nanosystems: application to microtubules and the ribosome. Proc Natl Acad Sci USA. (2001) 98:10037–41. 10.1073/pnas.181342398 11517324 PMC56910

[37] Wade RC Gabdoulline RR Rienzo FD. Protein interaction property similarity analysis. Int J Quantum Chem. (2001) 83:122–7. 10.1002/qua.1204

[38] Olsson MHM Søndergaard CR Rostkowski M Jensen JH. PROPKA3: consistent treatment of internal and surface residues in empirical pKa predictions. J Chem Theory Comput. (2011) 7:525–37. 10.1021/ct100578z 26596171

[39] Cornell WD Cieplak P Bayly CI Gould IR Merz KM Ferguson DM. A second generation force field for the simulation of proteins, nucleic acids, and organic molecules. J Am Chem Soc. (1995) 117:5179–97. 10.1021/ja00124a00215702930

[40] Hodgkin EE Richards WG. Molecular similarity based on electrostatic potential and electric field. Int J Quantum Chem. (1987) 32:105–10. 10.1002/qua.560320814

[41] Mallon DH Kling C Robb M Ellinghaus E Bradley JA Taylor CJ. Predicting humoral alloimmunity from differences in donor and recipient HLA surface electrostatic potential. J Immunol. (2018) 201:3780–92. 10.4049/jimmunol.1800683 30429288 PMC6287104

[42] Wu F Zhao S Yu B Chen Y-M Wang W Song Z-G. A new coronavirus associated with human respiratory disease in China. Nature. (2020) 579:265–9. 10.1038/s41586-020-2008-3 32015508 PMC7094943

[43] Kent WJ Sugnet CW Furey TS Roskin KM Pringle TH Zahler AM. The human genome browser at UCSC. Genome Res. (2002) 12:996–1006. 10.1101/gr.229102 12045153 PMC186604

[44] Trolle T McMurtrey CP Sidney J Bardet W Osborn SC Kaever T. The length distribution of class I restricted T cell epitopes is determined by both peptide supply and MHC allele specific binding preference. J Immunol Baltim Md. (2016) 196:1480–7. 10.4049/jimmunol.1501721 26783342 PMC4744552

[45] Mishto M Liepe J. Post-translational peptide splicing and T cell responses. Trends Immunol. (2017) 38:904–15. 10.1016/j.it.2017.07.011 28830734

[46] Alford RF Leaver-Fay A Jeliazkov JR O'Meara M DiMaio FP Park H. The rosetta all-atom energy function for macromolecular modeling and design. J Chem Theory Comput. (2017) 13:3031–48. 10.1021/acs.jctc.7b00125 28430426 PMC5717763

[47] Pickett BE Sadat EL Zhang Y Noronha JM Squires RB Hunt V. ViPR: an open bioinformatics database and analysis resource for virology research. Nucleic Acids Res. (2012) 40:D593–8. 10.1093/nar/gkr859 22006842 PMC3245011

[48] Janice Oh H-L Ken-En Gan S Bertoletti A Tan Y-J. Understanding the T cell immune response in SARS coronavirus infection. Emerg Microbes Infect. (2019) 1:1–6. 10.1038/emi.2012.26 26038429 PMC3636424

[49] Grifoni A Sidney J Zhang Y Scheuermann RH Peters B Sette A. A sequence homology and bioinformatic approach can predict candidate targets for immune responses to SARS-CoV-2. Cell Host Microbe. (2020) 27:671–80.e2. 10.1016/j.chom.2020.03.002 32183941 PMC7142693

[50] Thomsen MCF Nielsen M. Seq2Logo: a method for construction and visualization of amino acid binding motifs and sequence profiles including sequence weighting, pseudo counts and two-sided representation of amino acid enrichment and depletion. Nucleic Acids Res. (2012) 40:W281–7. 10.1093/nar/gks469 22638583 PMC3394285

[51] Rudolph MG Stanfield RL Wilson IA. How TCRs bind MHCs, peptides, and coreceptors. Annu Rev Immunol. (2006) 24:419–66. 10.1146/annurev.immunol.23.021704.115658 16551255

[52] Rossjohn J Gras S Miles JJ Turner SJ Godfrey DI McCluskey J. T cell antigen receptor recognition of antigen-presenting molecules. Annu Rev Immunol. (2015) 33:169–200. 10.1146/annurev-immunol-032414-112334 25493333

[53] Borrman T Cimons J Cosiano M Purcaro M Pierce BG Baker BM. ATLAS: A database linking binding affinities with structures for wild-type and mutant TCR-pMHC complexes. Proteins Struct Funct Bioinforma. (2017) 85:908–16. 10.1002/prot.25260 28160322 PMC5860664

[54] Riley TP Keller GLJ Smith AR Davancaze LM Arbuiso AG Devlin JR. Structure based prediction of neoantigen immunogenicity. Front Immunol. (2019) 10:2047. 10.3389/fimmu.2019.02047 31555277 PMC6724579

[55] Braun J Loyal L Frentsch M Wendisch D Georg P Kurth F. SARS-CoV-2-reactive T cells in healthy donors and patients with COVID-19. Nature. (2020). 10.1038/s41586-020-2598-9 32726801

[56] Grifoni A Weiskopf D Ramirez SI Mateus J Dan JM Moderbacher CR. Targets of T *Cell* responses to SARS-CoV-2 coronavirus in humans with COVID-19 disease and unexposed individuals. Cell. (2020) 181:1489–501.e15. 10.1016/j.cell.2020.05.015 32473127 PMC7237901

[57] Jurrus E Engel D Star K Monson K Brandi J Felberg LE. Improvements to the APBS biomolecular solvation software suite. Protein Sci. (2018) 27:112–28. 10.1002/pro.3280 28836357 PMC5734301

[58] The PyMOL Molecular Graphics System (version 1.7). Schrödinger, LLC.

[59] Nerli S Sgourakis NG. Structure-based modeling of SARS-CoV-2 peptide/HLA-A02 antigens. bioRxiv. (2020) 2020.03.23.004176. 10.1101/2020.03.23.004176 35047875 PMC8757863

