# Supplementary material for: Structure-Based Modeling of SARS-CoV-2 Peptide/HLA-A02 Antigens
Source: Front Med Technol. 2020 Nov 17;2:553478. doi: 10.3389/fmedt.2020.553478 (PMC8757863; doi:10.3389/fmedt.2020.553478)
Supplement: Supplementary file 6 [file Data_Sheet_1.PDF]

Supplementary Information

Structure-based modeling of SARS-CoV-2 peptide/HLA-A02 antigens

Santrupti Nerli[1] and Nikolaos G. Sgourakis[2,3]*

[1]Department of Biomolecular Engineering, University of California Santa Cruz, Santa Cruz, CA 95064, USA.
[2]Department of Chemistry and Biochemistry, University of California Santa Cruz, Santa Cruz, CA 95064, USA.
[3]Present address: Center for Computational and Genomic Medicine, The Children's Hospital of Philadelphia, and Department of Biochemistry and Biophysics, Perelman School of Medicine, University of Pennsylvania, 3401 Civic Center Blvd, Philadelphia, PA, 19104, USA
*To whom correspondence should be addressed.
E-Mail: Nikolaos.Sgourakis@Pennmedicine.upenn.edu

Supplementary Figures

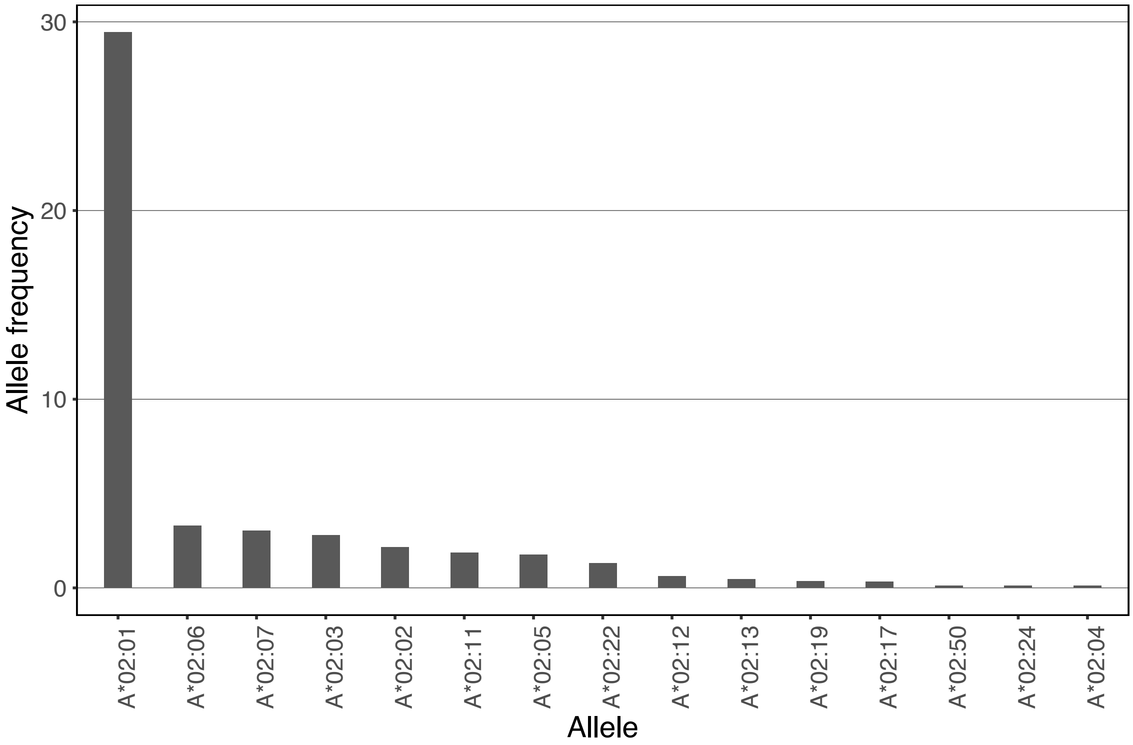

Supplementary Figure 1. Frequency of HLA-A02 alleles in world-wide population
Frequency (in %) of sub-type alleles of HLA-A02 super-type in world-wide population as reported in the Allele Frequency Net database (http://www.allelefrequencies.net) (Gonzalez-Galarza et al. 2020).

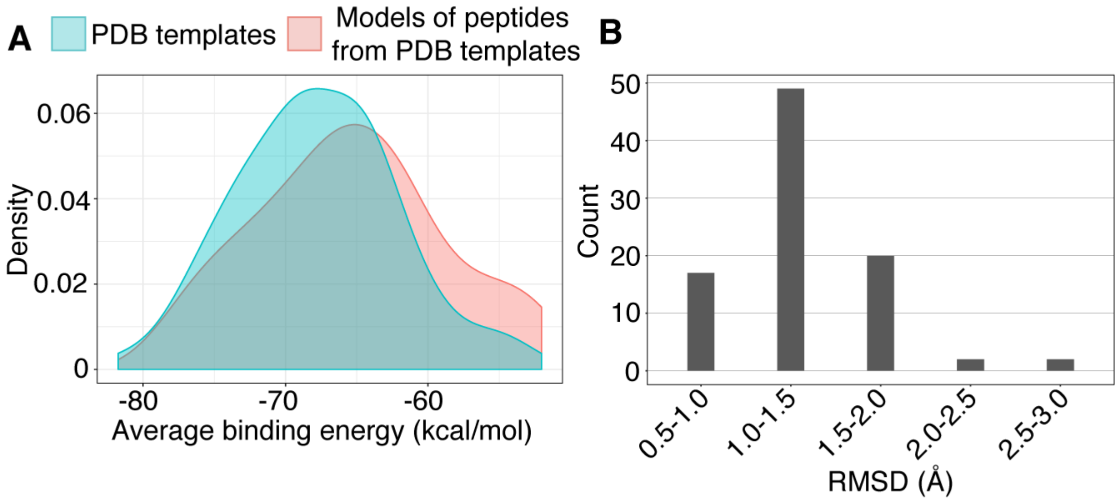

Supplementary Figure 2. Evaluating the accuracy of RosettaMHC using a benchmark set of 90 9mer/HLA-A02 crystal structures in PDB

(A) Density plots showing distribution of average Rosetta binding energies (kcal/mol) for 90 peptides of length 9 derived from HLA-A02 structures (or templates) in PDB (crimson) (Berman et al. 2000). The binding energies of relaxed PDB templates are shown by the cyan distribution. **(B)** RMSD (Å) distributions of the RosettaMHC models of the peptides derived from HLA-A02 structures in PDB compared with their native crystal structures.

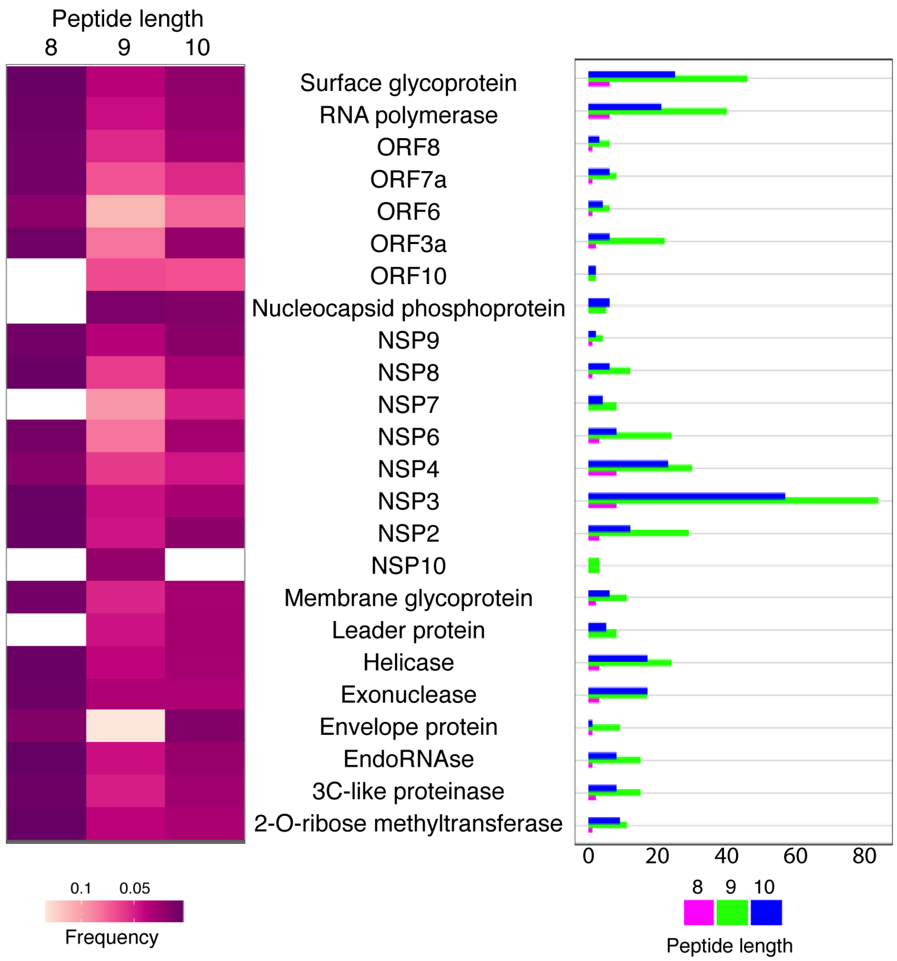

Supplementary Figure 3. Origin of SARS-CoV-2 epitopes predicted by NetMHCpan-4.0 (Left) Heat map showing the enrichment (Frequency) of NetMHCpan-4.0 (Jurtz et al. 2017) filtered epitopes of lengths 8, 9 and 10, in each of the SARS-CoV-2 protein. The color scale for the frequency is shown below the heat map. (Right) Bar plot showing a total number of NetMHCpan-4.0 filtered epitopes of lengths 8, 9 and 10 (magenta, green and blue, respectively) obtained from the SARS-CoV-2 proteins.

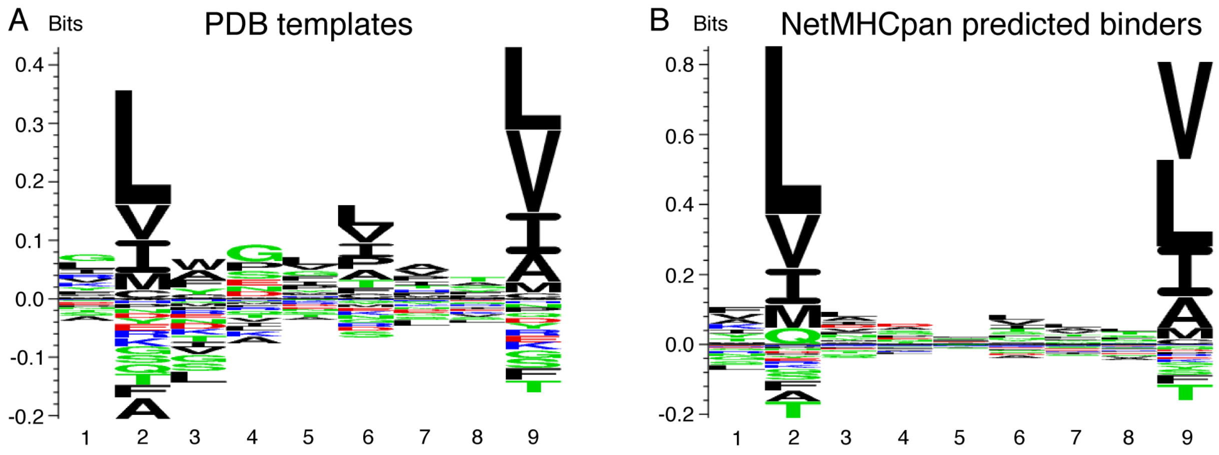

Supplementary Figure 4. Sequence motifs of peptides of length 9 from PDB templates and NetMHCpan-4.0 predicted SARS-CoV-2 binders

Sequence motifs of peptides of length 9 from **(A)** 90 HLA-A02 PDB templates and **(B)** 439 NetMHCpan-4.0 (Jurtz et al. 2017) predicted strong and weak binders selected based on default %rank cut-off values. The sequence logos were generated using Seq2Logo (Thomsen and Nielsen 2012).

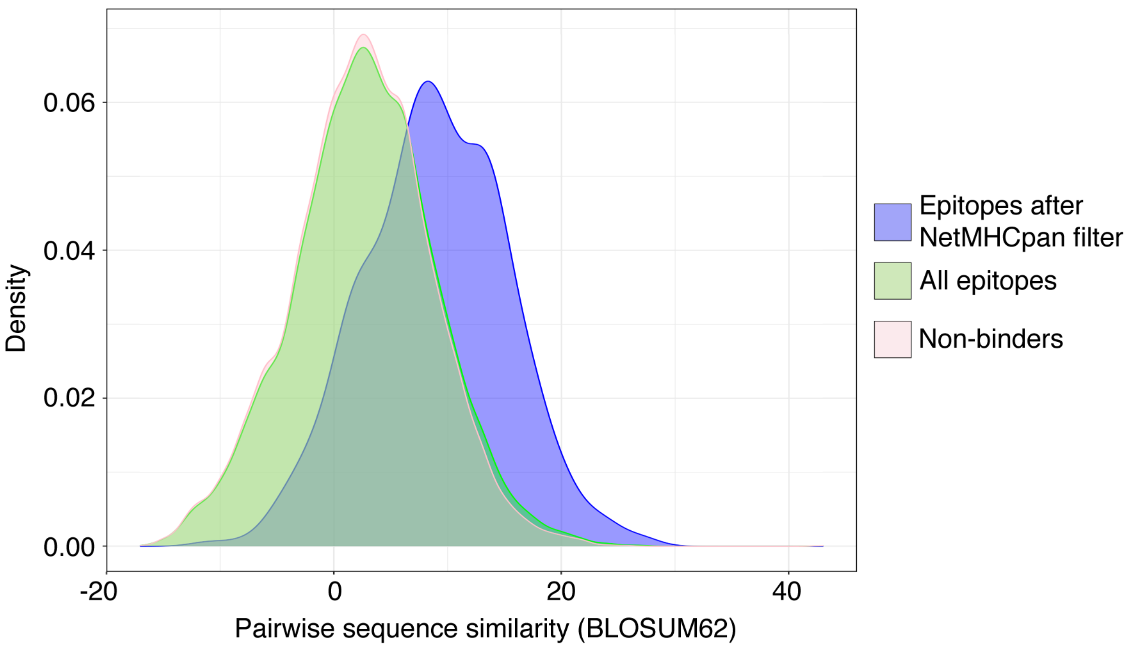

Supplementary Figure 5. Pairwise sequence similarity of the SARS-CoV-2 epitopes of length 9 with peptides derived from HLA-A02 structures in PDB

Distributions showing the pairwise sequence similarity in terms of BLOSUM62 (Henikoff and Henikoff 1992) score of the SARS-CoV-2 epitopes of length 9 and the corresponding peptide sequences selected as PDB templates for modeling. The sequence similarity score is shown for, SARS-CoV-2 epitopes before the NetMHCpan-4.0 filter (Jurtz et al. 2017) (All epitopes; green), the binding set after the NetMHCpan-4.0 filter (Epitopes after NetMHCpan-4.0 filter; blue) and the non-binding set after the NetMHCpan-4.0 filter (Non-binders; pink).

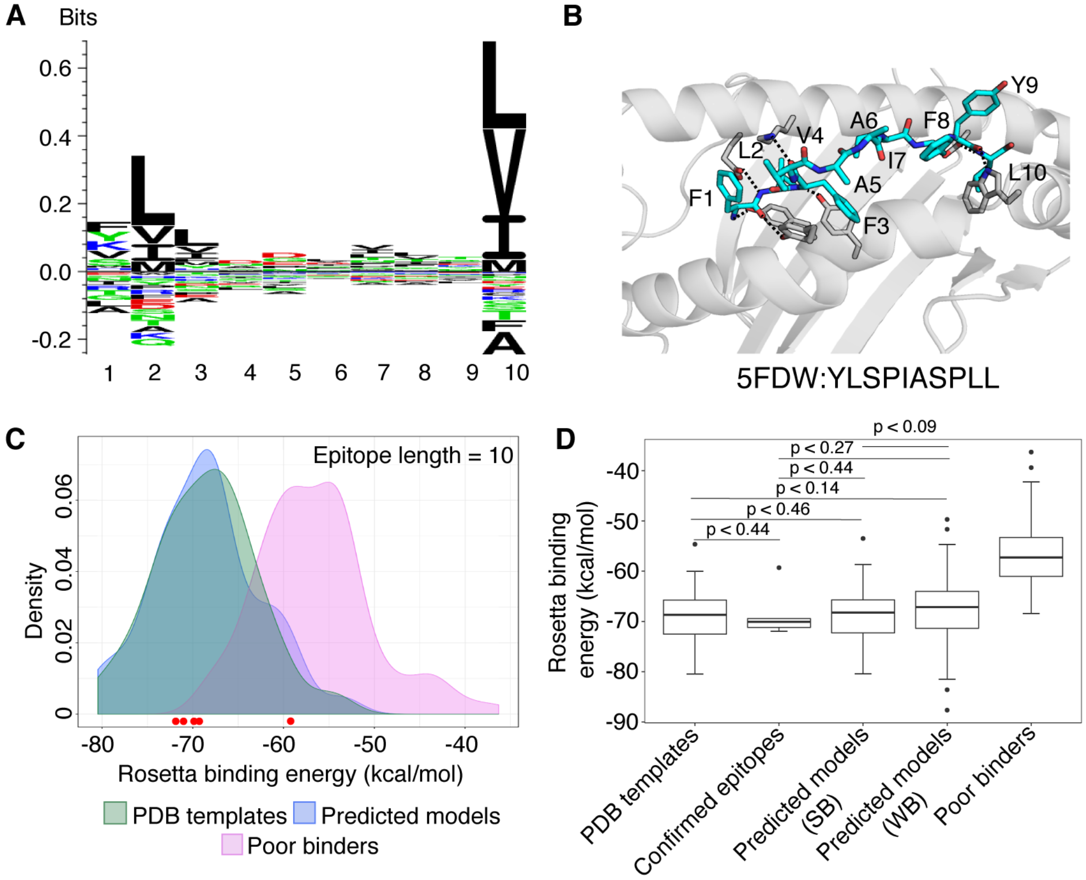

Supplementary Figure 6. RosettaMHC modeling results for SARS-CoV-2 epitopes of length 10

(A) Sequence logo from the 42 top ranking epitopes in the SARS-CoV-2 genome, predicted by NetMHCpan-4.0 (Jurtz et al. 2017). **(B)** The top 10mer epitope in our refined set, FLFVAAIFYL, from nsp4. Dotted lines indicate polar contacts between peptide and heavy chain residues, with peptide residues labelled. The template PDB ID and original peptide used for modeling the target peptide is indicated below the model. **(C)** Density plots showing distribution of average Rosetta binding energies (kcal/mol) for all epitopes of length 10. Distributions reflect 31 PDB templates (green), 42 strong binder epitopes (according to NetMHCpan-4.0 (Jurtz et al. 2017)) (blue), and 100 SARS-CoV-2 peptides classified as poor binders by NetMHCpan-4.0 modeled using the PDB templates and used as a reference set for sub-optimal binders (Poor binders; pink). The binding energies of models generated for 5 confirmed SARS T cell epitopes from the IEDB and ViPR (Grifoni et al. 2020; Pickett et al. 2012; Janice Oh et al. 2019) are indicated by circles at the bottom of the plot. Red circles indicate epitopes that lie within the distribution of refined PDB templates. **(D)** Box plots showing distribution of average binding energies for 31 PDB templates, 100 sub-optimal SARS-CoV-2 peptides, 5 confirmed epitopes (Grifoni et al. 2020; Pickett et al. 2012; Janice Oh et al. 2019) and RosettaMHC models for 42 strong (SB) and 214 weak (WB) binder 10mer epitopes predicted from the SARS-CoV-2 proteome using NetMHCpan-4.0. An unpaired Mann-Whitney U test was performed for relevant pairs of distributions and their statistical significance described by the *p*-values (where, $p < 0.1$ is considered statistically significant) are (i)

PDB templates and strong binders: $p < 0.46$ (ii) PDB templates and confirmed binders: $p < 0.44$ (iii) PDB templates and weak binders: $p < 0.14$ (iv) confirmed epitopes and strong binders: $p < 0.44$, and (v) confirmed epitopes and weak binders: $p < 0.27$, and (vi) strong and weak binders: $p < 0.09$ are shown inside the plot. The sequence logos were generated using Seq2Logo (Thomsen and Nielsen 2012).

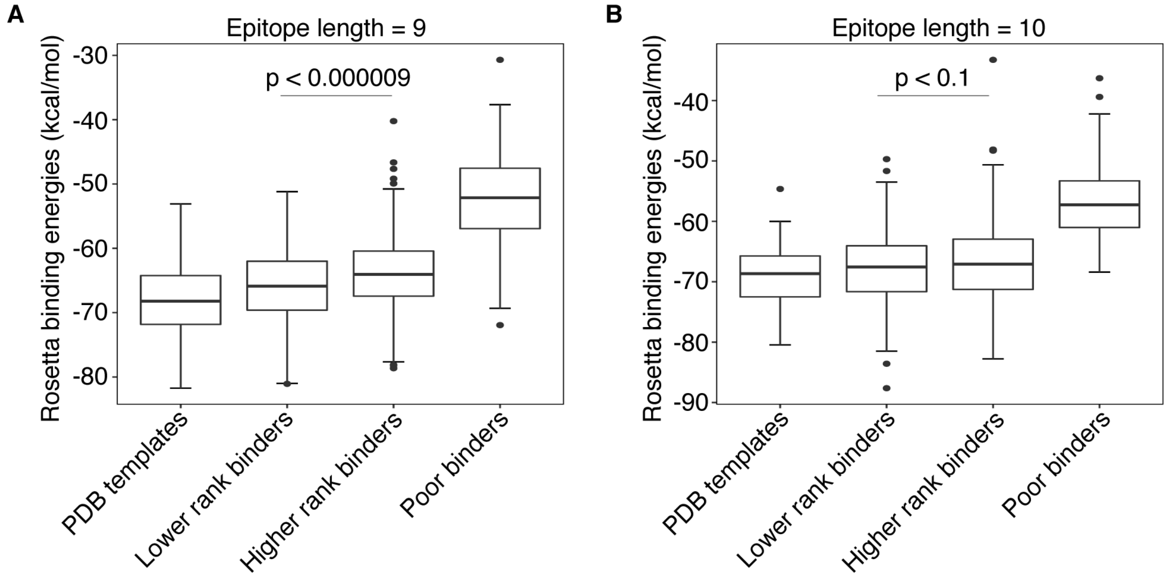

Supplementary Figure 7. Enrichment of the SARS-CoV-2 epitope set using higher NetMHCpan-4.0 %rank cut-off value

Box plots showing the distribution of average binding energies for 93 PDB templates, 100 sub-optimal SARS-CoV-2 peptides, and RosettaMHC models for **(A)** 439 9-mer epitopes with NetMHCpan-4.0 (Jurtz et al. 2017) %rank cut-off value of 2.0 (Lower rank binders or default) and 312 epitopes with %rank cut-off value of 4.0 and **(B)** 256 10-mer epitopes with %rank cut-off value of 2.0 (Lower rank binders or default) and 242 epitopes with %rank cut-off value of 4.0. An unpaired Mann-Whitney U test was performed for relevant pairs of distributions and their statistical significance described by the *p*-values for low and high rank binders of length (i) 9: $p < 0.000009$ (ii) 10: $p < 0.1$. The *p*-value < 0.1 for the test is considered statistically significant. The RosettaMHC models for higher ranked binders are available to the users upon request.

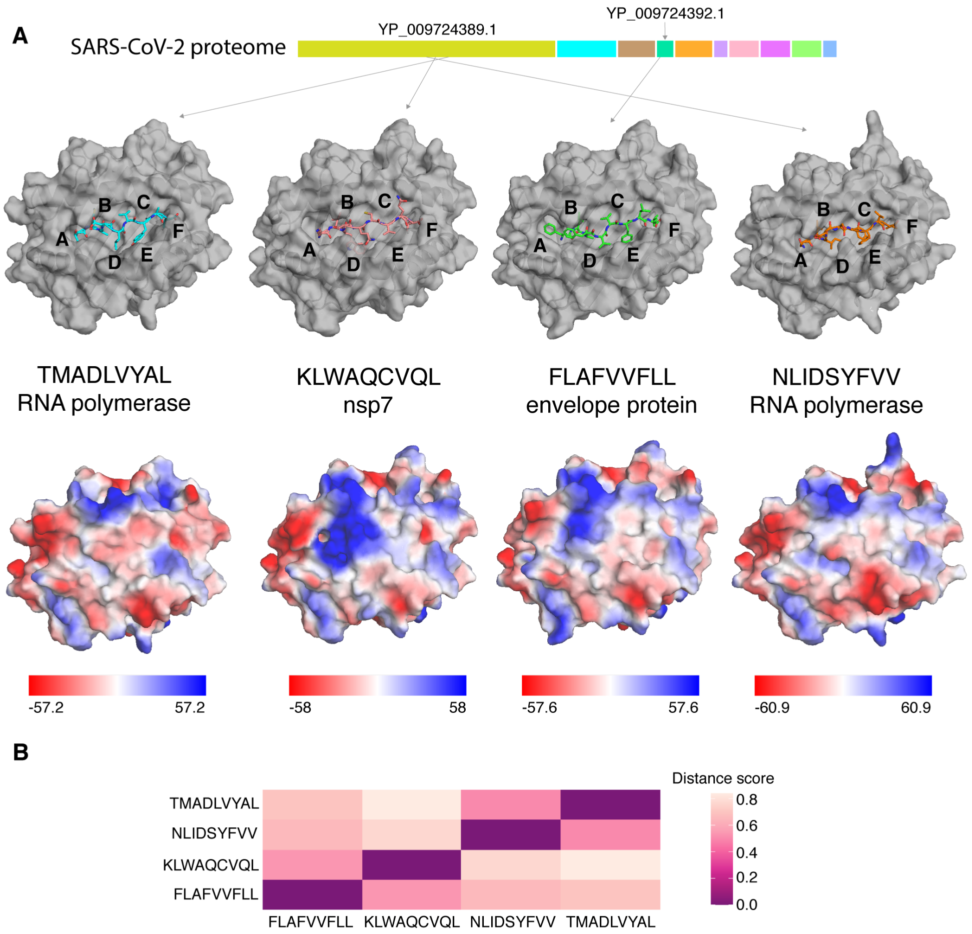

Supplementary Figure 8. Variability in TCR recognition surfaces of HLA-A02 with different high-affinity peptides. (A) Molecular surfaces of SARS-CoV-2/HLA-A*02:01 RosettaMHC models are shown for four top-scoring epitopes (ranked by Rosetta binding energy from left to right) captured in the A, B, C, D, E and F pockets of the MHC-I groove (top panel). The origins of the peptide epitopes in the ~30 kbp SARS-CoV-2 genome are noted. Electrostatic surfaces computed for the same models are shown in the bottom panel (Baker et al. 2001). Solvent-accessible surface representation with electrostatic potential in the indicated ranges (down to −61 kcal/(mol·e) in red and up to +61 kcal/(mol·e) in blue) were calculated using the APBS solver (Baker et al. 2001) in the mulitpipsa4.0.2 (Tong, Wade, and Bruce 2016) software. The visual representations of the electrostatic surfaces for the models are obtained from Pymol (*The PyMOL Molecular Graphics System* (version 1.7), n.d.). All calculations were performed at 150 mM ionic strength, 298.15 Kelvin, pH 7.2, protein dielectric 1.0, and solvent dielectric 78 with a probe radius of 1.4 Å. **(B)** A heat map showing electrostatic surface distance scores among four top-scoring epitopes listed in (A), computed using PIPSA (Wade, Gabdoulline, and Rienzo 2001; Tong, Wade, and Bruce 2016). A distance score key is shown on the right, where a score of 0 indicates electrostatic identity, 1 indicates no correlation and 2 indicates electrostatic anticorrelation.

Supplementary Tables

Supplementary Table 1: RosettaMHC binding energies of 749 SARS-CoV-2 epitopes of lengths 8, 9 and 10.

Supplementary Table 2: Confirmed SARS-CoV-2 epitopes homologous to SARS epitopes obtained from IEDB and ViPR databases (Grifoni et al. 2020; Janice Oh et al. 2019; Pickett et al. 2012).

Supplementary Table 3: Enriched SARS-CoV-2 epitope set of lengths 8, 9 and 10 obtained using higher NetMHCpan-4.0 %rank cut-off values.

Supplementary Table 4: Electrostatic surface distance scores of 254 SARS-CoV-2 epitopes of length 9 and their corresponding homologs from 229E, HKU1, NL63 and OC43 common cold coronavirus strains.

Supplementary Table 5: SARS-CoV-2 specific epitopes of length 9.

Supplementary Table 6: SARS-CoV-2 CD8+ cross-reactive T-cell epitopes known to induce immune responses in COVID-19 patients and healthy donors (Chour et al. 2020; Nelde et al. 2020).

Epitope	Homologous peptide sequences from common cold coronavirus strains	BLOSUM62 sequence similarity score[$]	Electrostatic surface distance score[#]
RLNEVAKNL	KLQTLIDNI (229E)	12	0.772
	LIQESIKSL (HKU1)	12	0.573
	ELQGLIDQI (NL63)	3	0.801
	RLQEAIKVL (OC43)	19	0.608
YLQPRTFLL	ALASYADVL (229E)	0	0.648
	PLSKRQYLL (HKU1)	15	0.463
	AFATFVDVL (NL63)	-5	0.703
	PLTSRQYLL (OC43)	14	0.506
FLHVTYVPA	FLHTVLLPT (229E)	25	0.564
	FMHFSYKPI (HKU1)	27	0.540
	FLHTVLLPT (NL63)	25	0.564
	FIHFSYVPT (OC43)	34	0.592
RLDKVEAEV	RLDTIQADQ (229E)	23	0.523
	RLDALEAQV (HKU1)	30	0.5
	RLDSIQADQ (NL63)	24	0.567
	RLDALEAEA (OC43)	29	0.522
LLFNKVTLA	ILFSKLVTS (229E)	19	0.51
	LLFDKVKLS (HKU1)	28	0.542
	LLFSKVVTS (NL63)	24	0.566
	LLFDKVKLS (OC43)	28	0.542

KIYSKHTPI	KGFSSDVLS (229E)	2	0.794
	YAYYADSGM (HKU1)	0	0.964
	NGYQHNSVV (NL63)	5	0.784
	YAYFTDTGV (OC43)	6	0.914

$ - BLOSUM62 sequence similarity score can range from negative to positive, where a higher score represents higher sequence similarity

# - Electrostatic surface distance scores between SARS-CoV-2 epitope/HLA complexes and common cold coronavirus epitope/HLA complexes were computed using PIPSA. Here 0, 1 and 2 indicate electrostatic identity, no correlation and electrostatic anticorrelation respectively.

Supplementary Methods

Extraction of SARS-CoV-2 epitopes

We created a single fasta file containing all the protein sequences of SARS-CoV-2, obtained from NCBI (https://www.ncbi.nlm.nih.gov/nuccore/NC_045512.2). To extract all the epitopes of specified length, we used *simple_sliding_window_epitope_creator* script available in the github under *covid_scripts* directory. An example command line is shown below.

(i) *python simple_sliding_window_epitope_creator.py –i [proteins.fasta] –l 9 > [epitopes.fasta]*

The above command line will generate all the epitopes of length 9 for the protein sequences in *proteins.fasta* file and outputs them into *epitopes.fasta* file.

Selection of SARS-CoV-2 epitope binders using NetMHCpan-4.0

As a first pass filter, we used stand-alone installation of NetMHCpan-4.0 (Jurtz et al. 2017) to select high-affinity SARS-CoV-2 epitopes. The NetMHCpan-4.0 software is available for download at https://services.healthtech.dtu.dk/software.php and the installation instructions are available at http://www.cbs.dtu.dk/services/doc/netMHCpan-4.0.readme. The NetMHCpan-4.0 software accepts as input a file containing peptide sequences and the allele name.

(i) *grep –v '>' [epitopes.fasta] > [epitopes.pep]*
(ii) *netMHCpan –p [epitopes.pep] > [netmhc_output.txt]*

The command specified in (i) is used to extract only the peptide sequences (*epitopes.pep*) from the fasta file format (*epitopes.fasta*). The command (ii) runs *netMHCpan* binary on the peptide sequences in the file *epitopes.pep* and outputs the classification into *netmhc_output.txt*. Here, we don't specify allele name since NetMHCpan-4.0 by default uses HLA-A*02:01. To extract only the strong and weak binders, we can use the following commands:

(iii) *grep –e "SB" –e "WB" [netmhc_output.txt] | awk '{printf ">%s\n%s\n",$10,$10}' > [epitopes_SB_WB.fasta]*

The corresponding epitopes classified as strong or weak binders are copied into *epitopes_SB_WB.fasta* file.

Structure modeling using RosettaMHC

RosettaMHC makes use of the protocols available in Rosetta (Leaver-Fay et al. 2011) via the wrappers written in PyRosetta (Chaudhury, Lyskov, and Gray 2010), to homology model peptides in a high-throughput manner. Specifically, it utilizes, Idealize, FastRelax (Tyka et al. 2011), Partial_thread and InterfaceAnalyzer protocols in its pipeline.

Idealize: This protocol is used to idealize the bond lengths and angles in the input template structure.

FastRelax: We used FastRelax (henceforth referred to as Relax) protocol with default parameters to carry out all-atom refinement of the peptide and the HLA binding groove (residues in the HLA molecule that are within a distance of 3.5 Å from the peptide). Relax combines side chain packing and gradient based minimization of the backbone degrees of freedom. The default parameters of Relax allows it to perform four rounds of side chain repacking followed by minimization of the backbone torsional angles. The repulsive energy term is tuned up to 2%, 25%, 55% and 100% in each of the four rounds.

Partial_thread: To thread a target sequence on to the template structure, we use partial_thread protocol.

InterfaceAnalyzer: The threaded structures are relaxed and the binding energies between peptide and the MHC molecules are extracted using the InterfaceAnalyzer protocol. Here, the dG_separated score term is reported as the binding energy. This term is computed by measuring the change in the energy when the peptide and the MHC chains are separated compared to their corresponding bound state structure.

RosettaMHC software is available for download at https://github.com/snerligit/mhc-pep-threader. This tool requires Python3, PyRosetta4, Biopython (Cock et al. 2009), and Clustal Omega (https://www.ebi.ac.uk/Tools/msa/clustalo/) (Sievers et al. 2011). The links to each of these tools is available on the README page of github.

After successful installation of the dependencies, the structural models of each peptide/HLA complex can be obtained using the following command:

(i) *python main.py –nstruct 10 –idealize-relax –relax-after-threading –template_pdb [3MRM.pdb] –mhcs [mhc_list] –peptides [pep_list] –mhc_chain A –peptide_chain P –pep_start_index 181 –interface_cutpoint 180*

In the above command line, the arguments (can be specified in any order) are used to indicate the following:

-*nstruct*: Number of independent relaxations (or optimizations) to perform. Here, 10 structural models are produced in the final stage of this protocol.
-*idealize-relax*: A flag to indicate to the program to idealize and relax the template PDB file before threading the target sequence on to the template structure.
-*relax-after-threading*: A flag to indicate to the program to relax the threaded structures.
-*template_pdb*: A template structure downloaded from the PDB. The user must download the PDB file and place it in the directory from which the above command is being executed.
-*mhcs*: An argument which accepts mhc_list file containing the allele name. For example, mhc_list file may contain one line: A*02:01.
-*peptides*: This is an argument which accepts a peptide list file in fasta format. In the above command line, pep_list is a fasta file containing peptide sequences.
-*mhc_chain*: Chain ID of the $\alpha 1$ and $\alpha 2$ sequences of the MHC molecule in the template PDB file.
-*peptide_chain*: Chain ID of the peptide sequence in the template PDB file.
-*pep_start_index*: When a target sequence is threaded on to a template structure, the threaded structure has only one chain consisting of both the peptide and the MHC molecule. To indicate where the peptide starts, this parameter is used. Typically, it is set to 181.
-*interface_cutpoint*: This argument specifies the last residue of the MHC molecule (we trim the MHC molecule to have only 180 residues in the $\alpha 1$ and $\alpha 2$ chains). This is where a single chain in the threaded structure is cleaved to create two separate chains for the interface analysis. This value is typically set to 180.

A *run.sh* script and an example is provided in the github under the directory examples/example-thread-peptide.

Selection of epitopes from common cold coronaviruses

We obtained sequences of *orf1ab*, *spike*, *envelope*, *membrane* and *nucleocapsid* proteins from each of the four strains of common cold coronavirus (229E, HKU1, NL63 and OC43) from NCBI.

(i) 229E: https://www.ncbi.nlm.nih.gov/nuccore/12175745
(ii) HKU1: https://www.ncbi.nlm.nih.gov/nuccore/85667876
(iii) NL63: https://www.ncbi.nlm.nih.gov/nuccore/49169782
(iv) OC43: https://www.ncbi.nlm.nih.gov/nuccore/1578871709

To identify the epitopes from common cold coronavirus strains that are homologous to SARS-CoV-2, we performed multiple sequence alignment of respective protein sequences using Clustal Omega. From the alignment, we extracted 395 epitopes of length 9 from all the strains that correspond to the NetMHCpan-4.0 predicted SARS-CoV-2 binders originating from 5 proteins of interest. Next, the common cold coronavirus epitopes are retained if (i) there are corresponding homologous epitopes in all the other strains and (ii) they do not contain any insertions or deletions.

After these steps, we found 254 common cold coronavirus epitopes in each strain that are homologous with SARS-CoV-2 epitopes of length 9. The scripts used to select common cold coronavirus epitopes are made available in the github under *covid_scripts* directory.

(i) *python identify_common_cold_peps.py -e [epitopes.txt] -aln [spike_glycoprotein.aln] -tag spike -l 9 -name HKU1 > [epitopes_HKU1_9.fasta]*

In the above command, *epitopes.txt* is a file containing high-affinity HLA-A02 peptide sequences from SARS-CoV-2 spike glycoprotein in each line. The file *spike_glycoprotein.aln* is an alignment file containing aligned sequences (from Clustal Omega) of spike protein from SARS-CoV-2 and all the other common cold coronavirus strains. The protein name and virus strain name along with peptide lengths are supplied using *tag*, *name* and *l* parameters.

The common cold coronavirus peptide/ HLA-A*02:01 complexes are modeled using RosettaMHC (Figure 1). A top scoring model from each epitope is retained for electrostatic analysis as described in the next section.

Electrostatic surface analysis of the RosettaMHC models

To perform the electrostatic surface analysis of the RosettaMHC models, we used multipipsa4.0.2 (Wade, Gabdoulline, and Rienzo 2001; Tong, Wade, and Bruce 2016). The multipipsa4.0.2 python wrapper internally utilizes PIPSA (Protein Interaction Property Similarity Analysis) protocol (Wade, Gabdoulline, and Rienzo 2001), which allows comparison of proteins using their electrostatic potentials computed using Adaptive Poisson-Boltzmann Solver (or APBS) (Baker et al. 2001). Below is the Python code we utilized to compare electrostatic surfaces of SARS-CoV-2 epitopes and peptides from common cold coronavirus strains.

run_pipsa.py

```
# specify the multipipsa installation directory and the pdbs directory containing all the PDB files
# that need to be analysed
localPIPSA = <multipipsa installation directory>
   examplePDBs = <directory containing the PDB files>
   print(localPIPSA)
   print(examplePDBs)

# below are the names of all the pdb files in the pdbs directory that you want to compare
   base = [<sars>]
   pdbfiles = [<229e>, <hku1>, <nl63>, <oc43>]
```

```
# align all the pdb files with <sars> pdb file
    stal = StructureAlign(structures=pdbfiles, dataDir=examplePDBs)
    stal.alignWithTemplate(template=base[0])

# fetch the names of the aligned pdb files
    aligned_pdbfiles = [<229e-aln>, <hku1-aln>, <nl63-aln>, <oc43-aln>]

# create APBS object and run APBS solver
    apbs = ApbsRun(dataDir=examplePDBs, pipsaRoot=localPIPSA,
              temp=298.15, pH="7.2", ios=0.150, structures= base+aligned_pdbfiles)
    apbs.runPdb2Pqr()
    apbs.runApbs()

# Create PIPSA object and run PIPSA over the entire protein
    print("\n\nRun plain PIPSA over total protein")
    pr = PipsaRun(pipsaRoot=localPIPSA,
              dataDir=examplePDBs,
              pointsTemplate=base[0])

    pr.setUseWeights(False)  # Optional
    pr.runPipsa(base+aligned_pdbfiles)

    os.system("<multipipsa install directory>/build/lib/multipipsa/data/pipsa/scr/do_pipsa_sim
<multipipsa install directory>/build/lib/multipipsa/data/pipsa/ "+ examplePDBs)
```

In the above code snippet, parameters within angular brackets (<>) must be replaced as required by the user's install and PDB directories and filenames. Upon execution of do_pipsa_sim, a sims.mat similarity matrix is created which contains distance measures between 0 (electrostatic identity) and 2 (electrostatic anticorrelation). All calculations were performed at 150 mM ionic strength, 298.15 Kelvin and pH of 7.2.

Supplementary Results

Enrichment of putative SARS-CoV-2 binding set using higher NetMHCpan-4.0 threshold

To enrich the putative binding set, we increased the NetMHCpan-4.0 %rank cut-off value to 4 compared to the default value of 2 and modeled the structures of additional 73, 312, and 242 epitopes of lengths 8, 9 and 10 respectively (Supplementary Table 3). The Rosetta binding energies of the enriched sets containing peptides of lengths 9 and 10 (higher %rank binders) fall between the average energies of the PDB templates and poor binders (epitopes classified by NetMHCpan-4.0 as worst binders) as shown in Supplementary Figure 7. Moreover, the energies of high ranked binders are comparable to the energies of peptides obtained using default %rank cut-off value (lower %rank binders) (even though the distributions are statistically significant; $p < 0.1$) which is likely due to the presence of favorable peptide anchor residues that contribute significantly to the energy measure (Toor et al. 2018). The structural models of 627 epitopes are available to users upon request from the authors.

Supplementary References

Baker, N. A., D. Sept, S. Joseph, M. J. Holst, and J. A. McCammon. 2001. "Electrostatics of Nanosystems: Application to Microtubules and the Ribosome." *Proceedings of the National Academy of Sciences of the United States of America* 98 (18): 10037–41. https://doi.org/10.1073/pnas.181342398.

Berman, H.M., J. Westbrook, Z. Feng, G. Gilliland, T.N. Bhat, H. Weissig, I.N. Shindyalov, and P.E. Bourne. 2000. "The Protein Data Bank." *Nucleic Acids Research* 28: 235–42. https://doi.org/10.1093/nar/28.1.235.

Chaudhury, Sidhartha, Sergey Lyskov, and Jeffrey J. Gray. 2010. "PyRosetta: A Script-Based Interface for Implementing Molecular Modeling Algorithms Using Rosetta." *Bioinformatics* 26 (5): 689–91. https://doi.org/10.1093/bioinformatics/btq007.

Chour, William, Alex M. Xu, Alphonsus H. C. Ng, Jongchan Choi, Jingyi Xie, Dan Yuan, John K. Lee, et al. 2020. "Shared Antigen-Specific CD8+ T Cell Responses Against the SARS-COV-2 Spike Protein in HLA A*02:01 COVID-19 Participants." *MedRxiv*, May, 2020.05.04.20085779. https://doi.org/10.1101/2020.05.04.20085779.

Cock, Peter J. A., Tiago Antao, Jeffrey T. Chang, Brad A. Chapman, Cymon J. Cox, Andrew Dalke, Iddo Friedberg, et al. 2009. "Biopython: Freely Available Python Tools for Computational Molecular Biology and Bioinformatics." *Bioinformatics* 25 (11): 1422–23. https://doi.org/10.1093/bioinformatics/btp163.

Gonzalez-Galarza, Faviel F., Antony McCabe, Eduardo J. Melo dos Santos, James Jones, Louise Takeshita, Nestor D. Ortega-Rivera, Glenda M. Del Cid-Pavon, et al. 2020. "Allele Frequency Net Database (AFND) 2020 Update: Gold-Standard Data Classification, Open Access Genotype Data and New Query Tools." *Nucleic Acids Research* 48 (D1): D783–88. https://doi.org/10.1093/nar/gkz1029.

Grifoni, Alba, John Sidney, Yun Zhang, Richard H. Scheuermann, Bjoern Peters, and Alessandro Sette. 2020. "A Sequence Homology and Bioinformatic Approach Can Predict Candidate Targets for Immune Responses to SARS-CoV-2." *Cell Host & Microbe*, March. https://doi.org/10.1016/j.chom.2020.03.002.

Henikoff, S., and J.G. Henikoff. 1992. "Amino Acid Substitution Matrices from Protein Blocks." *Proceedings of the National Academy of Sciences of the United States of America* 89 (22): 10915–19.

Janice Oh, Hsueh-Ling, Samuel Ken-En Gan, Antonio Bertoletti, and Yee-Joo Tan. 2019. "Understanding the T Cell Immune Response in SARS Coronavirus Infection." *Emerging Microbes & Infections* 1 (1): 1–6. https://doi.org/10.1038/emi.2012.26.

Jurtz, Vanessa, Sinu Paul, Massimo Andreatta, Paolo Marcatili, Bjoern Peters, and Morten Nielsen. 2017. "NetMHCpan-4.0: Improved Peptide-MHC Class I Interaction Predictions Integrating Eluted Ligand and Peptide Binding Affinity Data." *Journal of Immunology (Baltimore, Md.: 1950)* 199 (9): 3360–68. https://doi.org/10.4049/jimmunol.1700893.

Leaver-Fay, Andrew, Michael Tyka, Steven M. Lewis, Oliver F. Lange, James Thompson, Ron Jacak, Kristian W. Kaufman, et al. 2011. "ROSETTA3: An Object-Oriented Software Suite for the Simulation and Design of Macromolecules." *Methods in Enzymology* 487: 545–74. https://doi.org/10.1016/B978-0-12-381270-4.00019-6.

Nelde, Annika, Tatjana Bilich, Jonas S. Heitmann, Yacine Maringer, Helmut R. Salih, Malte Roerden, Maren Lübke, et al. 2020. "SARS-CoV-2 T-Cell Epitopes Define Heterologous

and COVID-19-Induced T-Cell Recognition.” *Research Square (Preprint)*, June. https://doi.org/10.21203/rs.3.rs-35331/v1.

Pickett, Brett E., Eva L. Sadat, Yun Zhang, Jyothi M. Noronha, R. Burke Squires, Victoria Hunt, Mengya Liu, et al. 2012. “ViPR: An Open Bioinformatics Database and Analysis Resource for Virology Research.” *Nucleic Acids Research* 40 (Database issue): D593–98. https://doi.org/10.1093/nar/gkr859.

Sievers, Fabian, Andreas Wilm, David Dineen, Toby J Gibson, Kevin Karplus, Weizhong Li, Rodrigo Lopez, et al. 2011. “Fast, Scalable Generation of High-Quality Protein Multiple Sequence Alignments Using Clustal Omega.” *Molecular Systems Biology* 7 (October): 539. https://doi.org/10.1038/msb.2011.75.

The PyMOL Molecular Graphics System (version 1.7). n.d. Schrödinger, LLC.

Thomsen, Martin Christen Frølund, and Morten Nielsen. 2012. “Seq2Logo: A Method for Construction and Visualization of Amino Acid Binding Motifs and Sequence Profiles Including Sequence Weighting, Pseudo Counts and Two-Sided Representation of Amino Acid Enrichment and Depletion.” *Nucleic Acids Research* 40 (Web Server issue): W281–87. https://doi.org/10.1093/nar/gks469.

Tong, Rudi, Rebecca C. Wade, and Neil J. Bruce. 2016. “Comparative Electrostatic Analysis of Adenylyl Cyclase for Isoform Dependent Regulation Properties.” *Proteins: Structure, Function, and Bioinformatics* 84 (12): 1844–58. https://doi.org/10.1002/prot.25167.

Toor, Jugmohit S., Arjun A. Rao, Andrew C. McShan, Mark Yarmarkovich, Santrupti Nerli, Karissa Yamaguchi, Ada A. Madejska, et al. 2018. “A Recurrent Mutation in Anaplastic Lymphoma Kinase with Distinct Neoepitope Conformations.” *Frontiers in Immunology* 9: 99. https://doi.org/10.3389/fimmu.2018.00099.

Tyka, Michael D., Daniel A. Keedy, Ingemar André, Frank Dimaio, Yifan Song, David C. Richardson, Jane S. Richardson, and David Baker. 2011. “Alternate States of Proteins Revealed by Detailed Energy Landscape Mapping.” *Journal of Molecular Biology* 405 (2): 607–18. https://doi.org/10.1016/j.jmb.2010.11.008.

Wade, R. C., R. R. Gabdoulline, and F. De Rienzo. 2001. “Protein Interaction Property Similarity Analysis.” *International Journal of Quantum Chemistry* 83 (3–4): 122–27. https://doi.org/10.1002/qua.1204.
